# Transcriptome analysis of adult *Caenorhabditis elegans* cells reveals tissue-specific gene and isoform expression

**DOI:** 10.1371/journal.pgen.1007559

**Published:** 2018-08-10

**Authors:** Rachel Kaletsky, Victoria Yao, April Williams, Alexi M. Runnels, Alicja Tadych, Shiyi Zhou, Olga G. Troyanskaya, Coleen T. Murphy

**Affiliations:** 1 Department of Molecular Biology, Princeton University, Princeton, New Jersey, United States of America; 2 Lewis-Sigler Institute for Integrative Genomics, Princeton University, Princeton, New Jersey, United States of America; 3 Department of Computer Science, Princeton University, Princeton, New Jersey, United States of America; 4 Flatiron Institute, Simons Foundation, New York, New York, United States of America; Stanford University School of Medicine, UNITED STATES

## Abstract

The biology and behavior of adults differ substantially from those of developing animals, and cell-specific information is critical for deciphering the biology of multicellular animals. Thus, adult tissue-specific transcriptomic data are critical for understanding molecular mechanisms that control their phenotypes. We used adult cell-specific isolation to identify the transcriptomes of *C*. *elegans’* four major tissues (or “tissue-ome”), identifying ubiquitously expressed and tissue-specific “enriched” genes. These data newly reveal the hypodermis’ metabolic character, suggest potential worm-human tissue orthologies, and identify tissue-specific changes in the Insulin/IGF-1 signaling pathway. Tissue-specific alternative splicing analysis identified a large set of collagen isoforms. Finally, we developed a machine learning-based prediction tool for 76 sub-tissue cell types, which we used to predict cellular expression differences in IIS/FOXO signaling, stage-specific TGF-β activity, and basal vs. memory-induced CREB transcription. Together, these data provide a rich resource for understanding the biology governing multicellular adult animals.

## Author summary

*C*. *elegans* is the simplest multi-cellular model system, with only 959 somatic cells in the fully-developed adult. This work describes the isolation and RNA-seq analysis of the worm’s major adult tissues. Previously, the isolation of adult tissues has been hampered by the worm’s tough outer cuticle, but identification of the transcriptomes of adult tissues is necessary to understand the biology of adults, which differs substantially from that of embryonic and larval cells. We recently developed a method to isolate and RNA-sequence adult tissues, and applied it here to characterize the muscle, neuron, intestine, and epidermis adult transcriptomes and isoform profiles. The data reveal interesting new characteristics for adult tissues, particularly the hypodermis’ metabolic function, which we have functionally tested. The tissue transcriptomes were also used to identify relevant human tissue orthologs in an unbiased manner. Finally, we present a new prediction tool for gene expression in up to 76 tissues and cell types, and we demonstrate its utility not only in predicting cell-specific gene expression, but in diagnosing expression changes in different genetic pathways and contexts.

## Introduction

Animals progress through many stages of development before reaching adulthood, and as adults, they exhibit metabolic and behavioral differences from developing animals. Studies in the nematode *C*. *elegans* demonstrate this phenomenon well: both biological responses and gene expression differ significantly in different stages [1,2]. Therefore, to understand the biology underlying tissue-specific adult behavior, it is critical to identify adult, tissue-specific transcriptomes.

The advent of whole-genome gene expression approaches allowed the identification of a cell’s full set of mRNA transcripts, ushering in a new era of understanding biological dynamics [3]. The ongoing development of new methods to isolate and sequence individual cells in order to approximate their metabolic and biochemical state has refined our understanding of single cells [4]. The next frontier in this work is the gene expression analysis of whole animals on a tissue-by-tissue and cell-by-cell basis. While tissue-specific expression has been measured in other organisms, the combination of extremely small tissue size and adult cuticle impermeability have previously prevented the analysis of adult worm tissue expression, which is necessary in order to understand adult processes, including systemic aging, tissue-specific aging, and cell non-autonomous control of aging. More broadly speaking, adult tissue-specific expression can be used to better understand signaling and cell autonomous processes and to compare expression to that in other adult organisms. The complexity of tissue autonomous and non-autonomous mechanisms of aging and disease requires the understanding of tissue-specific expression. The delineation of adult tissue expression presented here, combined with the genetic and molecular tools available in the worm, provide a unique chance to more directly model aging and disease compared to more complex organisms.

*C*. *elegans* is the simplest multicellular model system, with only 959 somatic (non-germline) cells in the fully developed adult animal. Four tissues—muscles, neurons, intestine, and the epidermis (or “hypodermis”)—comprise the bulk of the animal’s somatic cells and are largely responsible for the animal’s cell autonomous and non-autonomous biological regulation. Until recently, most transcriptional analyses of *C*. *elegans* adults utilized whole worms, but the need to identify tissue-specific transcripts in order to better understand both tissue-specific and non-autonomous signaling has become apparent. Several tissue profiling techniques that rely on PAB-mediated RNA immunoprecipitation have been widely used, but these methods often introduce very high non-specific background [5] and studies have not focused specifically on adult animals [1,6,7]. Recent spliced-leader RNA-tagging methods [8] that avoid this problem are also limited, since only 50–60% of *C*. *elegans* genes exhibit SL1-based trans-splicing [9]. Furthermore, tools used to isolate embryonic and larval stage *C*. *elegans* cells using cell sorting [1,10–13] have allowed the transcriptional profiling of specific tissues and cell types, shedding light on larval development processes, but lack information specific to adult tissues. Much of worm behavioral analysis, and all aging studies—for which *C*. *elegans* is a premier model system—[14] are, not is performed in adults, which are less amenable to standard isolation approaches due to their tough outer cuticle. Therefore, we developed a method to disrupt and isolate adult tissues [2]. That work revealed that the adult neuronal transcriptome differs significantly from earlier embryonic and larval stages, and that the adult neuronal transcriptome best reveals genes involved in behavior and neuronal function. The other major tissues—muscle, intestine, and hypodermis—are likely to provide insight into important adult-specific processes that are widely studied in *C*. *elegans* as models of human biology, such as pathogenesis, reproduction, age-related decline, and others.

Here we have performed cell-specific transcriptional analysis and characterization of the four major somatic tissues isolated from adult worms. As examples of the utility of these data, we used the highly enriched tissue gene sets to identify transcriptional parallels between worm and human tissues and to determine the tissue specificity of DAF-16 transcriptional targets. Additionally, our sequencing method allowed the identification of tissue-specific alternatively spliced gene isoforms, which we have used to explore tissue-specific collagen isoform expression. Finally, we present a tool that predicts gene expression in 76 different sub-tissue cell types, and demonstrate its utility in the characterization of individual genes, gene classes, and potential cellular differences in gene expression for several different signaling pathways. Together, these data provide a rich resource for the examination of adult gene expression in *C*. *elegans*.

## Results

### Isolation and sequencing of major adult *C*. *elegans* tissues

**Fig 1:**
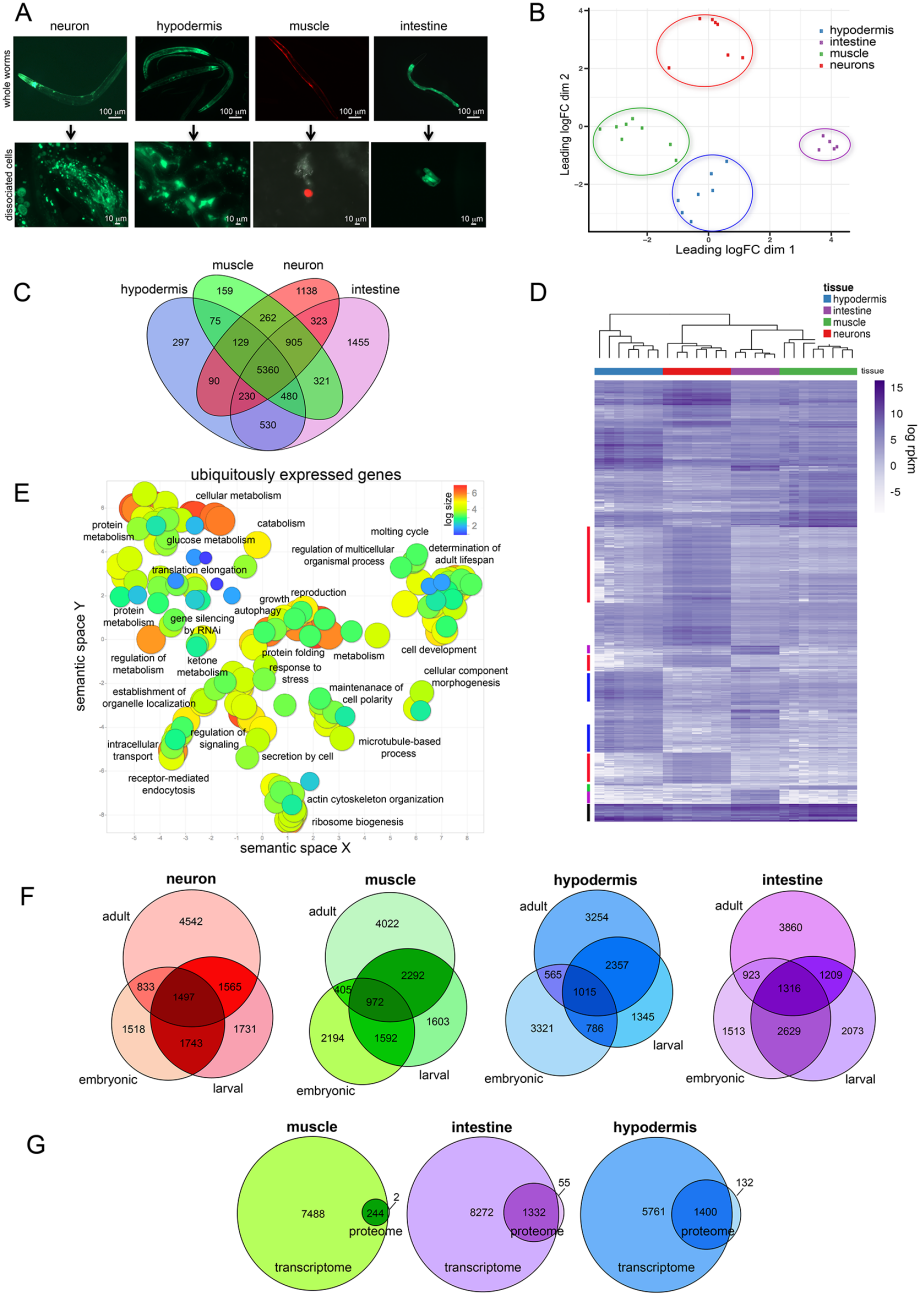
Sorting, RNA-seq, and analysis of isolated adult *C*. *elegans* tissues. A) Day 1 adult *C*. *elegans* strains used in our study to sort and identify neurons (*Punc119*::*GFP)*, hypodermis *(pY37A1B*.*5*::*GFP)*, muscle *(Pmyo-3*::*mCherry)*, and intestine (*Pges-1*::*GFP)* before (top) and after cell disruption (bottom, prior to debris filtering). B) Multidimensional scale plot of all samples used in this study. C) Venn comparison of expressed genes in each tissue. D) Heatmap of gene expression across tissues. Log rpkm expression values for the top 2000 differentially expressed genes per tissue (blue = high, white = low). Color bars (left) represent clusters of tissue specific gene expression (ubiquitous, black; hypodermis, blue; neuron, red; intestine, purple; muscle, green). E) Gene ontology (GO) analysis of ubiquitously expressed genes (defined as the 5,360 genes (see Venn above) expressed across all 4 tissues. GO terms with padj < 0.05 (hypergeometric test) were plotted using REVIGO [82]. Log size and was determined by the frequency of the GO term in the GO term database, where larger bubbles represent more general GO terms. Each label refers to an individual circle that is representative of the cluster of GO terms in its vicinity. F) Comparison of expressed genes in larval, embryonic [1], and adult for each tissue (neuron, muscle, hypodermis, intestine) with expression cutoffs as described in Spencer et al. [1]. G) Comparison of expressed genes with proteomic data in three adult tissues [21]. Proteins were classified as “expressed” if present in all (3 out of 3) biological replicates. Cytosolic and nuclear proteins were pooled for this comparison.

To identify the transcriptomes of adult *C*. *elegans* tissues, it is necessary to break open the outer cuticle and release, filter, and sort cells while minimizing cell damage [2]. We collected 27 Day 1 adult tissue samples (7 neuron, 5 intestine, 7 hypodermis, 8 muscle), utilizing strains with fluorescently-marked neurons (*Punc-119*::*gfp*), muscle (*Pmyo-3*::*mCherry*), hypodermis (*pY37A1B*.*5*::*gfp*), and intestine (*Pges-1*::*gfp*; Fig 1A; see Methods for details).

Multidimensional scaling analysis (Fig 1B) suggests that the samples cluster best with their respective tissue types, and that muscle and hypodermis are most closely related, while neuronal and intestine samples are more distinct from one another. Subsampling analysis [15], which determines whether sequencing has been performed to sufficient depth, suggests that this estimate of gene expression is stable across multiple sequencing depths (S1A Fig), and thus gene expression differences represent true differences between tissues.

We obtained reads across the whole transcript length (rather than selecting the 3’ end of mRNA via the polyA tail) in order to analyze tissue-specific isoform expression (see below). To assess RNA degradation in each sample, we determined the gene body coverage for all 20,389 protein-coding genes [16]; the transcripts have consistent, uniform coverage, with best coverage within the gene bodies (S1B Fig).

“Expressed” genes are defined as those with both (1) an average log(rpkm) greater than 2, and (2) with each replicate of that tissue having a log(rpkm) greater than 1, resulting in the detection of 8437 neuron, 7691 muscle, 7191 hypodermis, and 9604 intestine protein-coding genes (Fig 1C, S1 Table); 5360 genes are expressed in all sampled tissues. Hierarchical clustering of the top 2000 differentially-expressed genes per sample across the four tissue types shows that intra-group tissue samples are most similar, specific genes characterize particular tissue types (especially neurons), and that there is a subgroup of genes expressed in all tissues (Fig 1D). As expected, Gene Ontology (GO) analysis of the ubiquitously-expressed gene set shows that basic cell biological and metabolic processes are shared, including such terms as *intracellular transport*, *protein metabolism*, *catabolism*, *glucose metabolism*, *ribosome biogenesis*, *translation elongation*, *maintenance of cell polarity*, and *microtubule-based process* (Fig 1E; S2 Table). Additionally, terms associated with protection of the cell, such as *response to stress*, *autophagy*, *protein folding*, *gene silencing by RNAi*, and *determination of adult lifespan* appear in the ubiquitous category.

#### Adult tissues express a set of genes distinct from larval tissues

We previously observed that embryonic and larval transcriptomes lack information relevant to adult neuronal activity and behaviors [2]. The addition of four neuronal samples here reinforced this conclusion; more than four thousand transcripts were found in adult neurons that were not found in embryonic or larval analyses (Fig 1F), and those GO terms are related to neuronal function, while embryonic and larval genes [1] are associated with development [2] (S3 Table). Similarly, the hypodermis, muscle, and intestine all express transcripts that were not significantly detected in earlier life stages, including GO terms associated with *determination of adult lifespan* and other adult functions (S4–S6 Tables). Furthermore, in each adult tissue we see examples of highly expressed genes that have known associations with adult behaviors and functions, including reproduction, germline maintenance, mating, egg laying, and longevity. For example, adult-enriched genes include neuronal expression of neuropeptides (*nlp-18*, *-11*, *-14*, *-21*), FMRF-like peptides (*flp-1*, *-9–19*, *-28*, *-3*); synaptobrevin (*snb-1*), the sodium/potassium ATPase EAT-6, which regulates feeding, fertility, and lifespan [17], and *pdf-1*, which is involved in mate searching [18]. While collagen expression in the hypodermis is, not surprisingly, enriched across embryonic, larval, and adult stages, the adult hypodermis specifically expresses higher levels of lifespan regulators (*hsp-1*, *sip-1*, *dod-6*, and the 14-3-3 protein *ftt-2*), TGF-β pathway components involved in reproductive aging (*sma-2*, *-3*, *-4*, *-9*, *-10*), and metabolic enzymes (*mdh-1* and -*2*, *idh-1*, *enol-1*, *qdpr-1*, *tdo-2*). Intestinal adult targets include *abu-11*, which is induced by ER stress and whose overexpression increases lifespan [19], and *ril-1*, which regulates lifespan [20]. Several adult tissues express higher levels of secreted fatty acid and retinol-binding proteins (*far-* genes), specific ribosomal subunits, and cytoskeletal elements (e.g., *tni-3*, and act*-1*, *-2*). Previous embryonic and larval expression datasets lack many of these adult function genes and terms, underscoring the necessity of adult tissue-specific transcriptional analyses to describe adult biological functions. Conversely, comparison of the embryonic and larval tissue profiles to that of adults also reveals interesting biological components and pathways that are unique to development. For example, GO terms from genes unique to the embryonic hypodermis were highly enriched for G-protein coupled receptor signaling, including 88 GPCRs (FDR = 2.19e^-28^), while similar analysis of the unique larval genes showed enrichment of 40 nuclear hormone receptors (FDR = 2.27e^-4^).

#### Tissue-specific gene and protein expression largely overlap

Recently, Reinke, et al. (2017) used a proximity labeling-based proteomics method to identify ~3000 proteins in four large non-neuronal tissues of the worm, three of which (muscle, intestine, and hypodermis) are shared with our analysis [21]. We wondered how well these results correlate with our transcriptional data, given the differences in approaches. For the most part, our results agree, with the protein set comprising a subset of the genes identified in each tissue, while relatively few proteins are consistently detected only in the proteomic samples (Fig 1G). The overall size of each proteome is much smaller than the transcriptomes, likely due to the technical challenges of identifying tissue-specific proteins and the reduced sensitivity of the technique in some tissues, such as muscle [21]. The comprehensive, adult tissue transcriptomes are, therefore, a highly valuable resource for expression analyses, particularly for adult function and aging studies.

#### Tissue-enriched genes characterize individual tissues

**Fig 2:**
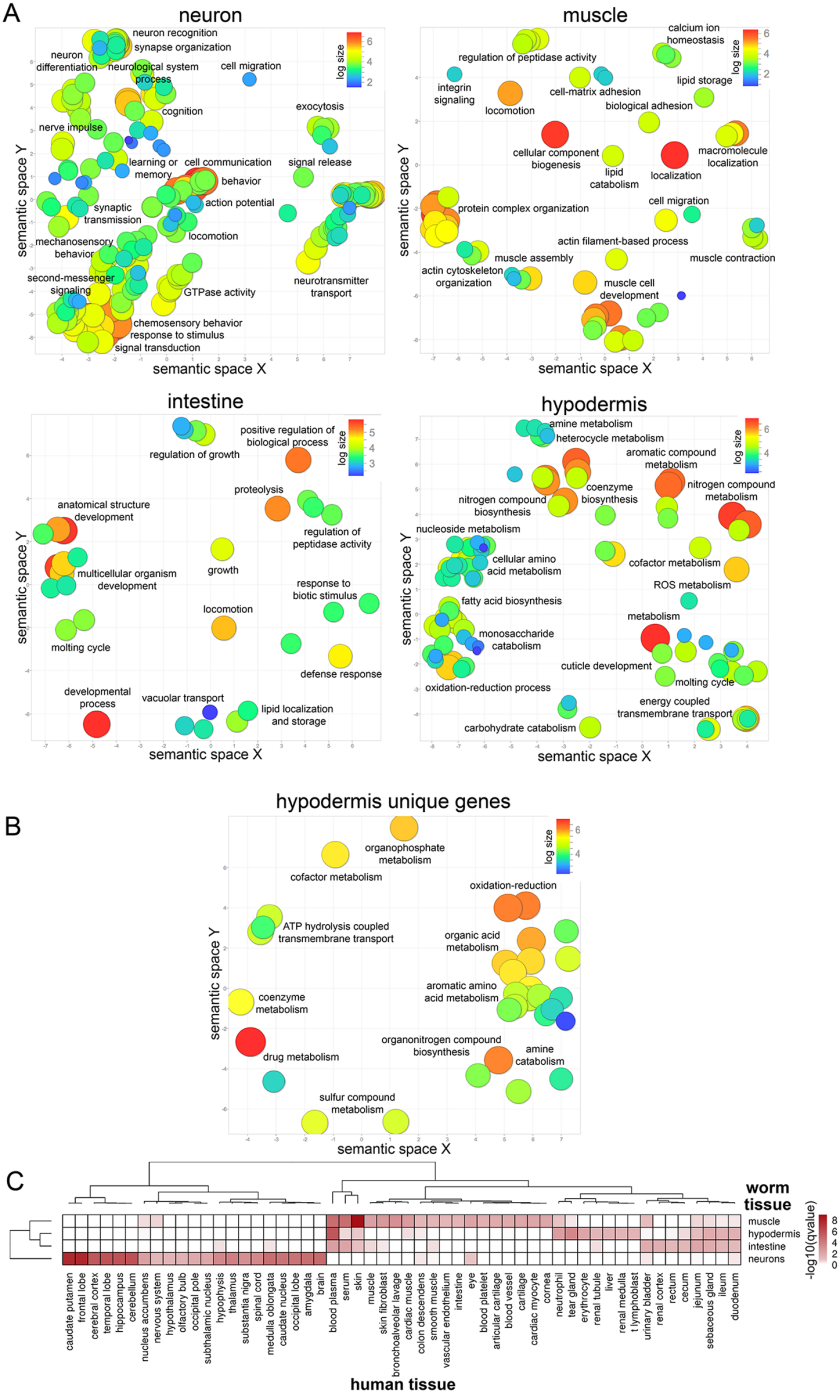
Characterization of tissue-enriched gene sets. A) Gene ontology analysis of each tissue-enriched gene list (S2 Table). GO terms with padj < 0.05 (hypergeometric test) were plotted using REVIGO. Log size and was determined by the frequency of the GO term in the GO term database, where larger bubbles represent more general GO terms. Each label refers to an individual circle that is representative of the cluster of GO terms in its vicinity. B) Gene ontology analysis of the hypodermis unique gene list (S9 Table). Log size and was determined by the frequency of the GO term in the GO term database, where larger bubbles represent more general GO terms. Each label refers to an individual circle that is representative of the cluster of GO terms in its vicinity. C) Comparison of each worm tissue (human orthologs of muscle, hypodermis, intestine, and neuron tissue-enriched gene lists) to human tissues (HPRD gene annotations [31]). Human tissues with q-value < 0.05 (hypergeometric test) were plotted (values plotted are −log_10_(q-value), white = not significant, dark red = highly significant enrichment).

We next identified the genes that were enriched in each tissue, that is, expressed at significantly higher levels in that tissue relative to all other tissues (FDR ≤ 0.05, log_FC_ > 2; S8 and S9 Tables; S2A Fig). By Spearman correlation analysis of tissue-enriched gene expression (S2B Fig), neurons are the most distinct tissue. The GO terms for neurons and muscle (Fig 2A, S2 Table) are largely expected: muscle’s tissue-enriched genes are associated with *muscle contraction*, *actin filament-based process*, *cell migration*, *calcium ion homeostasis*, *actin cytoskeleton organization*, *integrin signaling*, etc., while the neuronal tissue-enriched set is associated with many well-characterized neuronal functions, including *learning or memory*, *synaptic transmission*, and *neurotransmitter transport* (Fig 2A, S2 Table). GO analysis of the intestine suggested that in addition to expected categories associated with digestion (*peptidase activity*, *proteolysis*), response to bacteria (*defense response*, *response to biotic stimulus*), and lipid metabolism (*localization* and *storage*), terms associated with the molting cycle, driven by the high expression of several collagens, appeared in the intestine tissue-enriched gene list (Fig 2A, S2 Table).

The *C*. *elegans* hypodermis is a relatively uncharacterized tissue, best understood for its role in collagen expression of the cuticle and molting during development [22], and those GO terms are represented here (Fig 2A, S2 Table). However, it is interesting to note that the vast majority of GO terms associated with the hypodermal tissue-enriched genes are related to different types of metabolism, including c*arbohydrate*, *amino acid*, *fatty acid*, *monosaccharide*, *nucleoside*, *ROS*, *co-factor*, *nitrogen compound*, *aromatic compound*, and *heterocycle metabolism*, as well as *co-enzyme biosynthesis* and *oxidation-reduction process*. The hypodermis unique genes are similarly enriched for metabolic processes (Fig 2B), and the promoters of the unique genes are enriched for the ELT-3 motif (p value = 2.09x10^-4^), which is itself a hypodermis unique gene (S9 Table). These data suggest that the hypodermis, a syncytial tissue that makes up a substantial fraction of the adult animal’s body, may be a major site of metabolic processes in *C*. *elegans*. Indeed, knockdown of several hypodermal-enriched or hypodermal-unique genes alter lipid levels (S2C and S2D Fig), and the hypodermal unique genes also include regulators of amino acid, drug, co-factor, ATP and organic acid metabolism (Fig 2B).

Motif analysis of the enriched genes for each tissue identified several candidate tissue-specific transcription factors (TFs) (S10 Table). Hypodermal genes were enriched for binding sites of known hypodermal TFs, including ELT-3, BLMP-1, and NHR-25, and both the hypodermis and intestine tissue-enriched genes showed significant overrepresentation of TGATAAG, the DAF-16 Associated Element, which PQM-1 binds [23]; all four of these transcription factors are in our hypodermis expressed list, while ELT-3 is unique to the hypodermis (S1 and S9 Tables). The intestine was enriched for motifs associated with EGL-27 and ELT-7, while muscle was enriched for JUN-1, DMD-6, LIN-29, and ZTF-19/PAT-9. Neuron-enriched motifs include SKN-1/Nrf2, whose activity in neurons regulates longevity [24,25], and MBR-1 (honeybee Mblk-1 related factor), which is expressed in the AIM interneuron, the site of long-term memory formation [26,27]. Several additional tissue-enriched and tissue-unique transcription factors (S8 and S9 Tables) provide further insight into the tissue-specific regulation of gene expression. For example, several well-documented examples of tissue-specific TFs are present, including the muscle enrichment of *unc-120*, involved in muscle maturation and maintenance, and the unique neuronal expression of *unc-86* that is required for the cell fate of several neuronal lineages. The tissue-specific expression of uncharacterized TFs (S8 and S9 Tables) may reveal new roles for transcriptional regulation in tissue development and maintenance.

#### Identification of orthologous worm/human tissues

*C*. *elegans* can be used as a model to study human disease due to its high degree of genetic, proteomic, cellular, behavioral, and tissue conservation. Functional comparisons are often used to draw parallels between worm and human tissues; for example, fat storage in the worm intestine is comparable to the function of human adipose tissue [28], and the worm hypodermis, forming an outer protective layer covered in cuticle, has been used as a model for human skin [29]. Using our tissue-specific sequencing data, we compared transcriptional profile similarities of worm and human tissues. In an unbiased approach, we identified the human orthologs [30] of genes in our tissue-enriched gene lists and compared them with curated tissue-specific gene annotations from the Human Protein Reference Database [31] for significant overlap (hypergeometric test). Not surprisingly, worm neurons are transcriptionally most similar to various human brain regions and neuronal tissues, and worm muscle is similar to human muscle (Fig 2D, S11 Table). Worm muscle also corresponded with human skin, sharing genes with GO terms related to basement membranes and cell migration, such as laminins, osteonectin, and integrins. Worm muscle and human skin also shared genes related to retinoic acid binding proteins (*lbp-1*, *-2*, *-3*/ CRABP1 and 2) (S11 Table). The worm intestine showed correlation with human small and large intestine, as well as parts of the human kidney, including the urinary bladder and renal cortex, sharing genes involved in the renin-angiotensin system (*acn-1*/ACE2, *asp-3*/REN) and various solute carrier family proteins. Surprisingly, the worm hypodermis exhibited no significant similarity to human skin, but rather correlated with liver and blood plasma, driven largely by metabolic genes (e.g., *ahcy-1*, *sams-1*, *cth-2*; S11 Table). Hypodermis also correlated with immune cells, such as neutrophils and T cells, potentially related to the immune functions of the worm hypodermis (Fig 2D) [32]; the worm hypodermis and human neutrophils share genes involved in microbial responses such as *ZC416*.*6*/LTA4H (leukotriene A4 hydrolase), *sta-2*/STAT3, *adt-2*/CFP (ADAMTS metalloprotease/complement regulator), *ftn-2*/FTL (iron metabolism), and *ctl-2*/CAT (catalase) [33–37]. These cross-species tissue similarities may be useful in developing new *C*. *elegans* models of human disease.

#### Adults express tissue-specific isoforms

**Fig 3:**
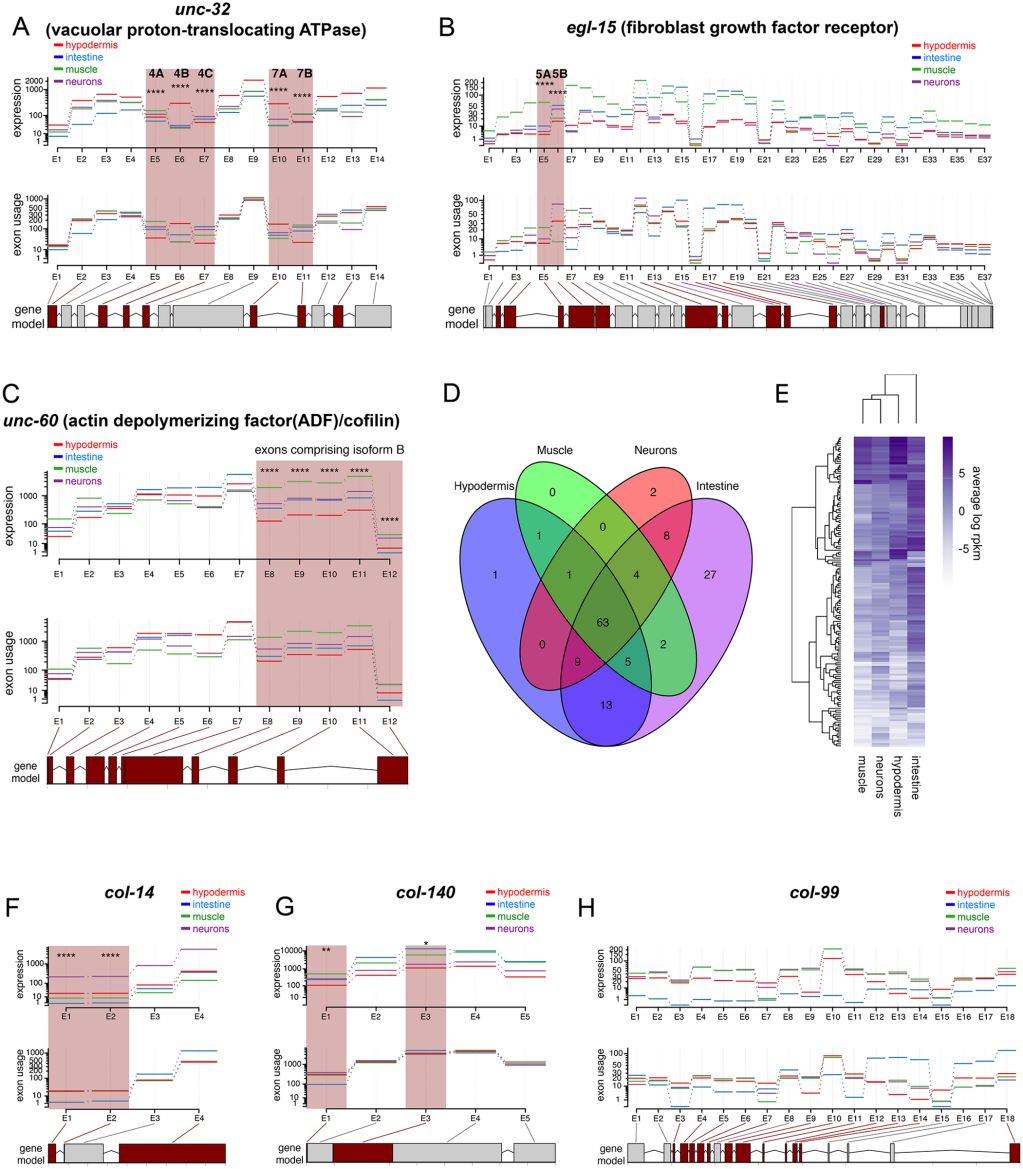
Alternative splicing analysis. A-C) DEXseq was used to identify significant splicing differences among tissues. Alternative splicing of *unc-32* (A), *egl-15* (B), and *unc-60* (C) across tissues shown as examples that correlate well with previous reports. D) Comparison of collagen expression (S1 Table) across the major adult tissues. E) Heatmap of collagen expression across adult tissues (average log rpkm; blue = high; white = low). F-H) Collagens are expressed in multiple tissues, and some are spliced in a complex manner. * p<0.05, ** p<0.01, *** p<0.001, **** p<0.0001.

Previously, global tissue-specific isoform expression has been difficult to assess because most experiments have used whole worms, have detected gene expression using isoform-indiscriminant microarray platforms, require affinity purification [38], or only utilize short reads biased toward the 3’ ends of mRNA [10]. Here we used relatively long reads with good coverage through most gene bodies (S1B Fig), and thus were able to assess the levels of expression of individual exons in each tissue. We identified 23,087 exons with significant tissue-specific alternative splicing (S12 Table). Several well documented tissue-specific splicing events are corroborated by our data; for example, *unc-32*, a vacuolar proton-translocating ATPase, exhibits mutually exclusive exon splicing at two separate locations (exons 4 A/B/C and 7 A/B) [39], and our data support the selective neuronal enrichment of exons 4B and 7A compared to other tissues (Fig 3A). Other examples include *egl-15*’s muscle-specific inclusion of exon 5A and repression of exon 5B [40] (Fig 3B), and the ubiquitous expression of *unc-60* isoform A compared to the muscle-specific enrichment of isoform B [41] (Fig 3C).

We previously found that collagens play an unexpected but important role in the regulation of lifespan [42]. Our expression data reveal that collagens are expressed not just in the hypodermis as anticipated, but also in neurons, muscle, and intestine (Fig 3D and 3E); additionally, a surprising number of collagens are alternatively spliced, exhibiting tissue-specific isoform expression (e.g., *col-14*; Fig 3F). While most collagens have relatively simple exon structures, including those with differential splicing such as *col-140* (Fig 3G), some are complex; for example, *col-99* has at least 18 exons, with isoform expression in hypodermis, intestine, muscle, and neurons (Fig 3H). The role of tissue-specific collagen isoforms offers an interesting avenue for further study.

#### Case studies for the utility of tissue-specific gene expression

In multicellular organisms, tissue- and cell-type specificity underlie pathway-level interactions between genes. To understand the precise function of genes and pathways, as well as disease (which results from their dysregulation), it is thus crucial to determine tissue-specific gene expression. Furthermore, tissues detect information from the environment and coordinate responses by communicating to other tissues using chemical and mechanical signals. By examining the tissue-expression patterns of various receptor classes, transcription factors, secreted molecules, and components of signaling pathways, a more complete understanding of autonomous and non-autonomous tissue signaling can be achieved.

#### Case study 1: Expression of signaling-related genes across adult tissues

**Fig 4:**
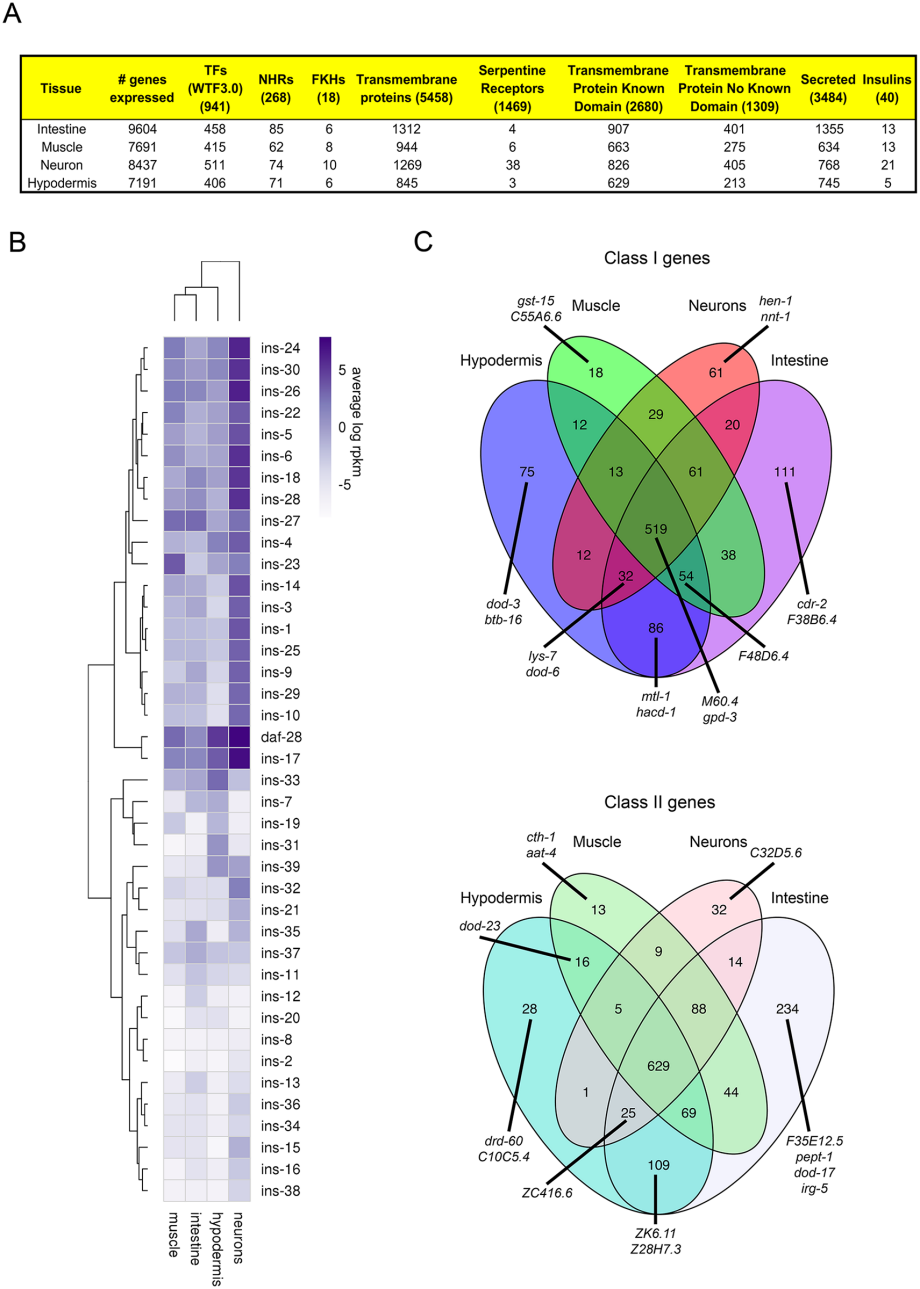
Tissue specificity of gene expression. A) Classes of genes expressed in each tissue type; original gene category lists from [43,83,84]. TF, transcription factors; NHR, nuclear hormone receptors; FKH, forkhead transcription factor family. B) Heatmap of insulin expression across adult tissues (average log rpkm; blue = high; white = low). C) Class I (upregulated in long-lived *daf-2* worms, Tepper et al. 2013 at 5% FDR, top), and Class II (downregulated, bottom) tissue expression Venn diagrams show that 45–47% of previously identified targets are shared among all tissues. The highest fraction of Class I and II targets are expressed in the intestine and hypodermis. Examples of genes in each category, including the top 10 genes ranked in the previously published IIS/FOXO data [23] are shown.

To start to understand activity within and communication between tissues, we examined the expression of specific gene classes (Fig 4A). The expression of transcription factors, including nuclear hormone receptors, is evenly distributed across tissues, while neurons have the highest counts of serpentine receptors (Fig 4A, S7 Table), perhaps unsurprising given their role in interpreting and communicating the environmental state to the rest of the animal. Neurons and intestine express a high number of transmembrane proteins, and the intestine also expresses the highest number of secreted proteins, suggesting roles in intercellular communication.

#### Case study 2: Insulin-like gene expression

The *C*. *elegans* genome encodes at least 40 insulin-like genes [43]; while some of these insulins have been studied for their roles in communicating nutritional, environmental, and behavioral status to the rest of the animal [44], the function of many insulins remains unknown. While insulin genes are expressed most highly in neurons, they can be found in every tissue, including hypodermis (Fig 4A and 4B), consistent with previous reports [43,45,46]. The further characterization of the tissue specificity of these factors will be helpful in building models to describe how the worm’s adult cells communicate with one another.

#### Case study 3: IIS/FOXO target expression in adult tissues

One of the applications of our tissue-specific transcriptional information is to identify tissue-specific targets of genetic pathways that affect both autonomous and non-autonomous gene expression. As an example of how one could use these data, we have approximated target tissue specificity of the Insulin/IGF-1 signaling (IIS) pathway from whole-worm expression data. IIS is one of the major longevity regulation pathways, controlling the nuclear translocation and subsequent transcriptional activity of the DAF-16/FOXO transcription factor [47,48]. IIS acts both cell autonomously and non-autonomously to regulate lifespan [49–51], highlighting the need to distinguish the two sets of regulators. The whole-worm downstream targets of DAF-16 have been identified and many have been characterized [23,52]. While the tissue-specific expression of a few of these targets is known [2,53], tissue-specific information is not available for the entire set of somatic targets.

Utilizing the canonical ranked list of IIS/DAF-16 targets [23] at a 5% FDR cutoff, we determined the site of expression of these Class I and II targets (*daf-2* up- and down-regulated, respectively) in our tissue-specific expressed gene sets. The largest subset (34%) of IIS/DAF-16 target genes are expressed in all tissues (e.g., *gpd-3*, *M60*.*4*), echoing the *adult determination of lifespan* category in the GO analysis of the ubiquitous gene set (Figs 4C and 1E). 63% of the Class I and II targets are expressed in the intestine, which has been previously identified as a site of DAF-16 regulation [23,50]; 10% are expressed exclusively in intestine (e.g., *cdr-2*, *pept-1*, *dod-17*; Fig 4C, S13 Table). Fifty percent of the targets are expressed in the hypodermis, with about 13% expressed exclusively there (e.g., *dod-3*, *btb-16*, *drd-50*; 3%) or shared just in the intestine and hypodermis (e.g., *mtl-1*, *hacd-1*, *ZK6*.*11*; another 10%). These data support the role of the hypodermis as a major site of insulin signaling and DAF-16 activity [53]. The most highly expressed hypodermal gene, the Notch ligand *osm-11*, is secreted by the hypodermis, and its knockdown leads to a DAF-16-dependent lifespan increase [54]. Another top Class I target, the mitochondrial protein *icl-1*/*gei-7*, is essential for the desiccation tolerance [55] and lifespan extension [52] of *daf-2* mutants. Hypodermal genes from this list can affect lipid storage in the intestine; knockdown of *pmt-1* leads to an increase in lipid droplet size and an increase in *fat-7* levels, as is observed in *daf-2* animals [56].

There are relatively few muscle-specific Class I or II DAF-16 targets (0.9%), and muscle subsets are all relatively sparse compared to the intestine and hypodermis. This may correlate with the observation that while DAF-16 expression in muscle rescues reproductive span [57], it does not rescue lifespan or dauer formation, unlike DAF-16 expression in intestine and neurons [50]. Class I and II neuron-specific targets include *hen-1* and *nnt-1*, which are both highly regulated by the IIS pathway [2,23,52]. These findings are consistent with the documented and predicted (see below) expression patterns for Class I and Class II genes (Fig 4C, S8 Fig). Together, these data start to better delineate the tissue-specific functions of the IIS/DAF-16 pathway. A similar approach can be taken with any mutant or pathway for which whole-animal gene expression data are available.

#### Genome-wide expression predictions span 76 tissues and cell types

**Fig 5:**
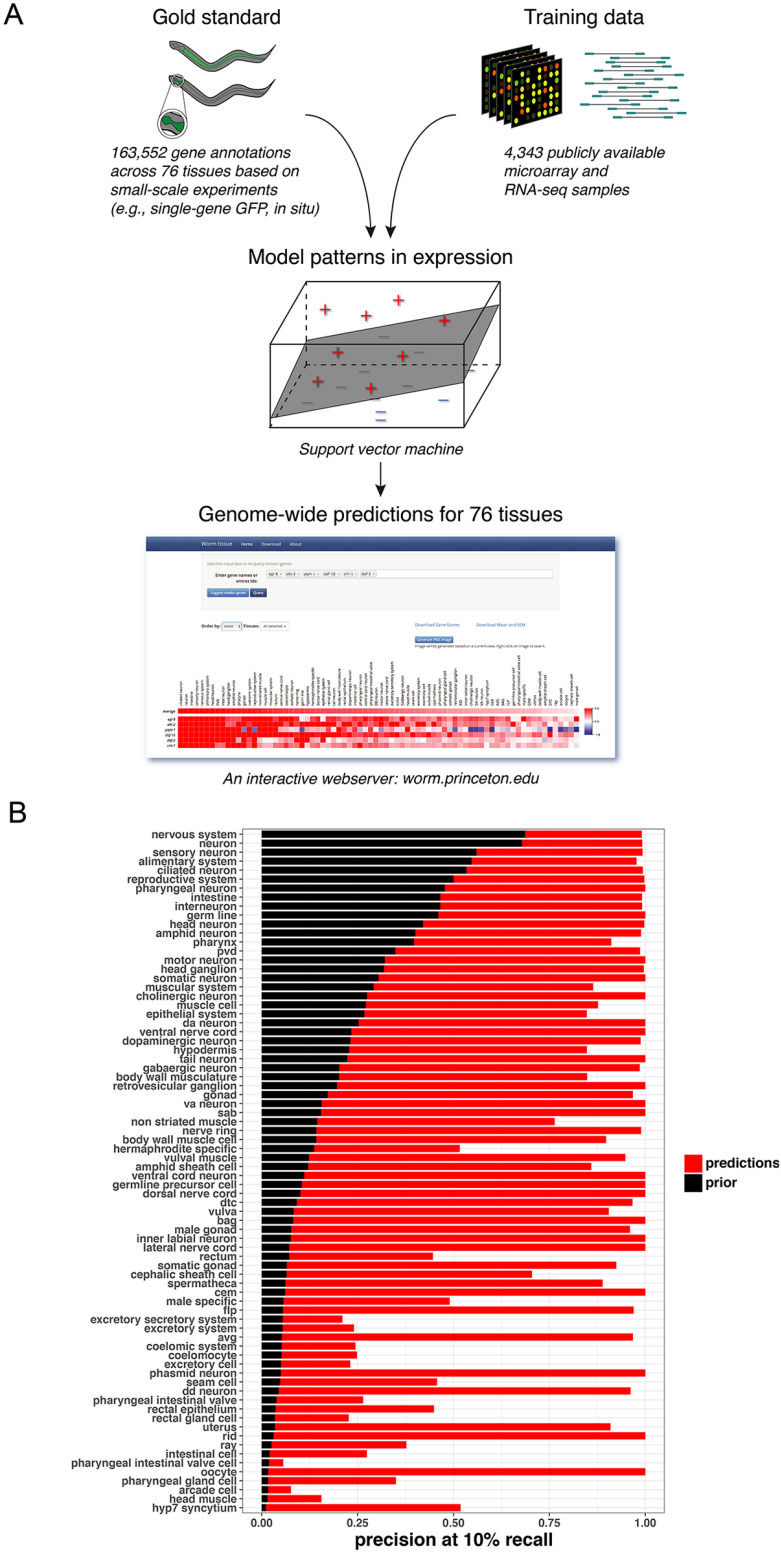
New prediction tool for sub-tissue/cellular gene expression. A) Overview of the tissue expression prediction tool. We obtained 163,552 curated gene annotations to tissue expression based on small-scale experiments from WormBase and the *C*. *elegans* Tissue Expression Consortium (gold standard) and collected 4,372 microarray and RNA-seq samples (training data). For each of 76 tissues and cell types, we integrated the gold standard and training data using a support vector machine to make genome-wide predictions. Finally, we built an interactive webserver (worm.princeton.edu), where users can explore and download these predictions. B) Prediction performance of tissue expression models. Precision at 10% recall based on 5-fold cross-validation is shown. High precision is achieved even for tissues and cell types where there are relatively few known examples of tissue-specific expression based on small-scale experiments (low prior, where prior is the expected fraction of positive predictions if genes were chosen at random).

To create a more complete and high resolution picture of spatiotemporal gene expression for tissues and life stages not yet characterized by direct sequencing analysis, we combined experimentally isolated tissue datasets with computational models of tissue-specific gene expression to generate genome-wide predictions across 76 diverse *C*. *elegans* tissues and cell types. This builds upon our previous machine learning approach that predicts gene expression across a few major tissues [5]. Briefly, the key insight of our method is that tissue-specific responses and expression patterns can be observed even in whole-animal expression data. Although the vast majority of publicly available expression data is from whole animals, we can leverage their sheer quantity (4,372 microarray and RNA-seq experiments across 273 datasets, including the adult "tissue-ome" presented above; Fig 5A). Using this expression compendium as training data in concert with a high quality gold standard (163,552 annotations based on small-scale experiments, i.e., genes known to be expressed in a particular tissue) within a machine learning framework allows us to model patterns of tissue-specific gene expression and make predictions of tissue- and cell-type specific expression (across 76 tissues and cell types) for every gene in the genome, even for genes that have never been profiled (in high throughput or small-scale experiment) in that tissue or cell-type (Fig 5A).

To examine the quality of our predictions, we examined prediction performance using five-fold cross-validation, where the known gene annotations are split into five random partitions, and each partition is completely hidden from the algorithm as it is trained on the other four. Evaluating against the hidden annotations, we observed impressive performance: median precision of 0.968 at 10% recall, where maximum possible of a perfect classifier is 1, (Fig 5B; and S3–S5 Figs). Notably, several tissue predictions perform strongly even with few gold standards in the training set, including touch receptor neurons, phasmid neurons, and oocytes (Fig 5B).

These predictions greatly expand upon the limited set of genes known to be expressed (gold standards) for each tissue subtype, and can provide insight into a variety of biological processes. For example, the dopaminergic (DA) neurons are widely studied for their roles in behavior modulation and neurodegeneration [58]. Using the novel genes predicted to be expressed in DA neurons at 10% recall, we find Gene Ontology enrichment of genes related to neuropeptide signaling and thigmotaxis (the use of surface textures as a navigational cue). Interestingly, thigmotaxis in *C*. *elegans* was recently shown to require dopamine signaling [59], and some the predicted DA genes may contribute cell autonomously to that process.

**Fig 6:**
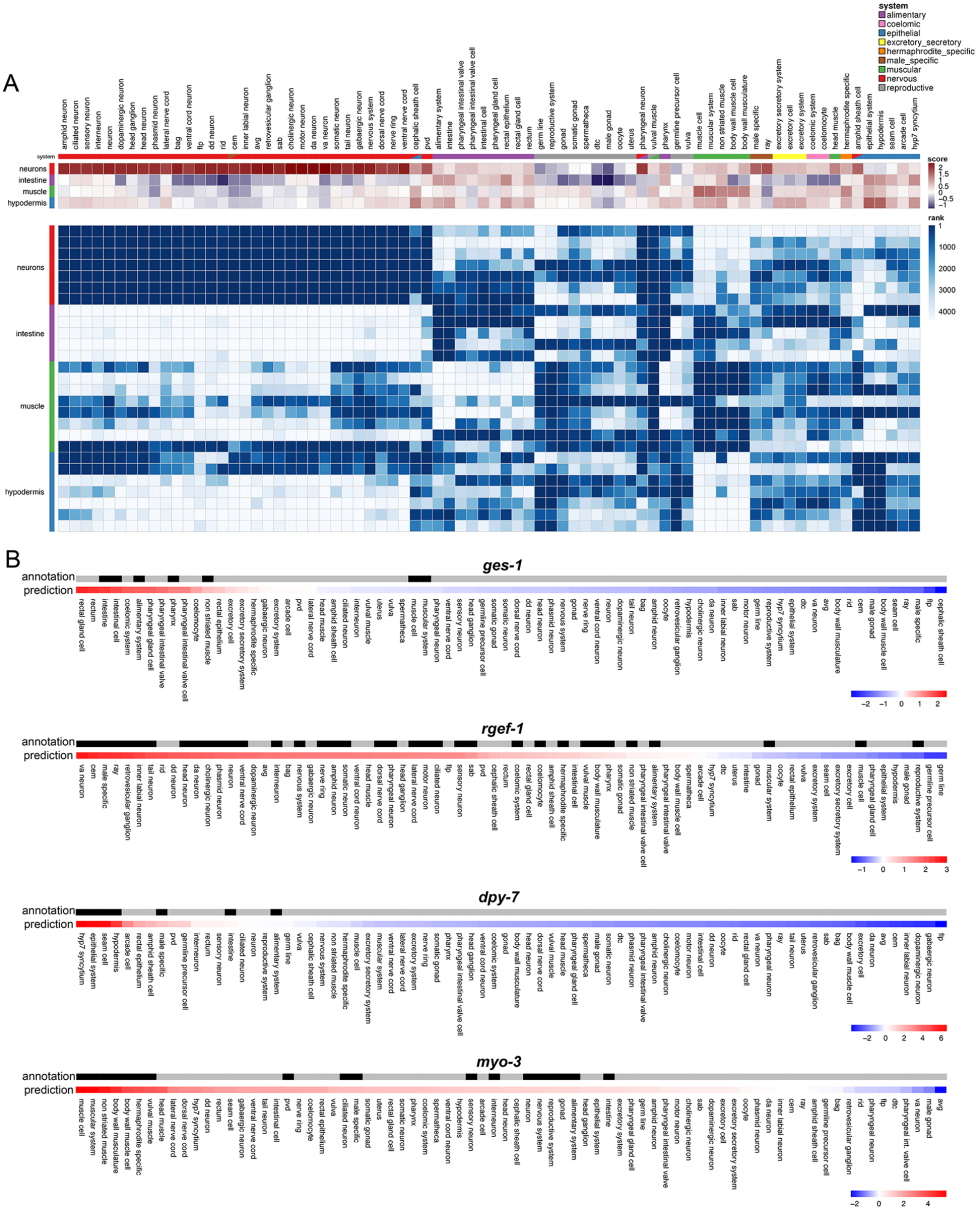
Tissue specificity of Insulin signaling/DAF-16 target expression. A) Top: Concordance between predicted tissue expression scores and tissue-ome (isolated tissue RNA-seq) samples. Each row represents one of 4 tissue-ome tissues for which tissue-enriched genes were identified, and each column represents one of 76 tissues for which predictions were made. Columns were hierarchically clustered, and the tissue system(s) for each tissue is indicated. Each cell represents average predicted tissue expression (normalized SVM) score for corresponding tissue-enriched genes Bottom: Contribution of tissue-ome samples to tissue-gene prediction models. Each row represents one of 27 tissue-ome samples (across 4 tissues), and each column represents one of 76 tissues for which predictions were made. Row and column orderings are as in A. Each cell represents rank of sample in contribution to the corresponding tissue model. B) Wormbase curated gene annotations are shown in black for *ges-1*, *rgef-1*, *dpy-7*, and *myo-3*. Below the annotations are the predictions for each gene, where red corresponds to the highest prediction score and blue is the lowest prediction score.

To further evaluate the adult “tissue-ome” (Fig 1), we generated predictions excluding our tissue-specific RNA-seq samples from the data compendium, then calculated average predicted tissue expression scores for each tissue’s enriched genes (S2 Table) across the 76 cell types. The predictions accurately characterized the identity of each sequenced tissue (Fig 6A). The predicted profiles in the 35 nervous system-related tissue expression models are especially striking, with very distinct, strong signals for the neuron tissue-enriched genes.

We also explored the contribution of the tissue-ome to machine learning models trained using the full data compendium. Taking advantage of the fact that for linear support vector machine (SVM) models, larger feature weights indicate higher relevance to the model [60], we examined the relative importance of each sample in the tissue-ome dataset. As expected, tissue samples were strongly upweighted in their relevant tissue models (Fig 6A) when compared to the other 4,345 samples in our data compendium.

As a proof-of-principle for predicting individual gene expression using this tool, we compared the gold standard annotations to the predictions for several commonly studied ‘tissue specific’ genes. The machine learning predictions largely recapitulate the known expression of *ges-1*, *rgef-1*, *dpy-7*, and *myo-3* (Fig 6B), further validating the prediction capabilities across various cell types.

#### Applying the prediction tool

Individual and group gene expression predictions can be made using our interactive website (http://worm.princeton.edu) (Fig 5A). In addition to predicted expression, the tool also displays gold standard annotations from literature, allowing the user to visualize expression across many cell types (Fig 6B). Predictions can also be downloaded, enabling their use for any additional analyses, such as in enrichment calculations for tissue or cell-type expressed genes, thus expanding the power of tools that currently rely solely on documented expression annotations (e.g., [61]).

Here, we used the tool to predict the site of expression of the 40 insulins (S6B, S7A and S7B Fig), revealing primarily expression in neurons, but also specific expression in non-neuronal tissues, such as germline, gonad, and male-specific tissues. We also examined several IIS pathway regulators to demonstrate the relevance of single gene predictions. Analysis of *daf-2* and *daf-16* predicts largely ubiquitous tissue distribution (S6A Fig), consistent with published data [48,62] and the expression observed in the four major adult tissues (S1 Table), as well as cell-autonomous functions of IIS in various tissues. The IIS/Class II gene-associated TF *pqm-1* [23] exhibits broad expression, including in some neuronal subtypes and additional cells (S6A Fig, S1 Table), suggesting previously untested roles for this pathway.

To demonstrate the biological insight that can be gained from cell type expression prediction analysis of large gene sets, we analyzed published data sets of IIS/DAF-16, TGF-β, and CREB signaling, as examples. For the IIS Class I and Class II targets, the tool recapitulates known patterns, such as intestinal and neuronal expression, and also suggests new expression patterns in tissue sub-types (S8A and S8B Fig).

The tool can be used to distinguish and characterize transcription targets of a single pathway at different developmental stages, as well. TGF-β Sma/Mab pathway signaling is highly conserved in worms and mammals, and regulates many diverse phenotypes including reproductive aging, body size, immune responses, and aversive olfactory learning [63–66]. The neuron-derived ligand DBL-1 modulates TGF-β transcription factor activity in the hypodermis, intestine, and pharynx, while hypodermal TGF-β signaling non-cell autonomously regulates oocyte quality underlying reproductive span [57]. Not surprisingly, these oocyte target genes are enriched for expression in oocytes and the reproductive system, as well as ubiquitously expressed genes (S9A Fig). By contrast, whole worm *sma-2* target genes from L4 animals lacking oocytes [57] are depleted for predicted reproductive system genes and are highly enriched in both predicted hypodermal and neuronal genes (S9B Fig).

**Fig 7:**
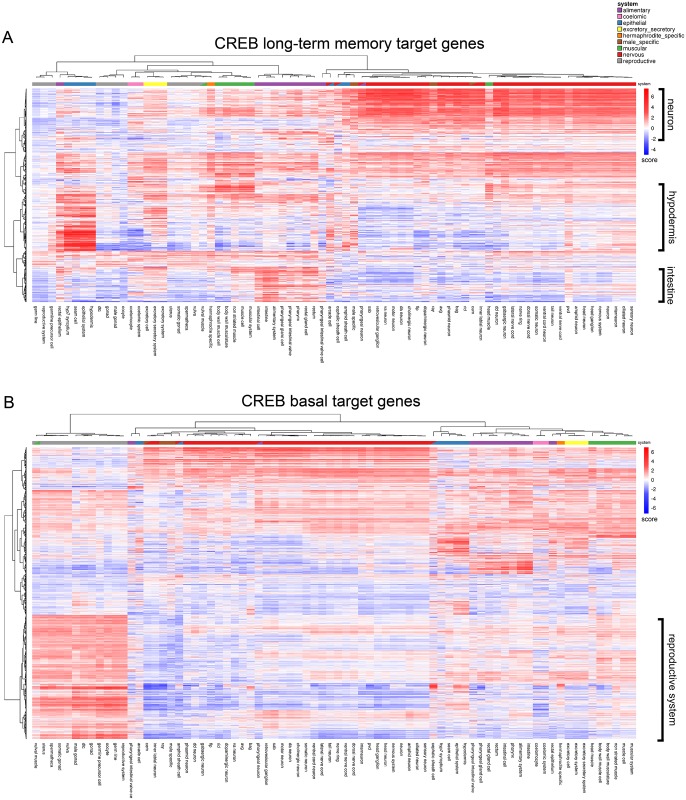
CREB-dependent long-term memory (LTAM) genes (A) and CREB-dependent naïve (basal) target genes (B) from Day 1 adult worms [27] were analyzed for predicted tissue expression. Heatmaps were plotted using the NMF R package [85].

Finally, we examined the transcriptional targets of CREB, which regulates both metabolism [67], and long-term memory in mammals and *C*. *elegans* [68,69]. The tissue-specific role of CREB in non-neuronal tissues remains less well explored despite its broad tissue expression in *C*. *elegans* (Fig 7A, S1 Table) and mammals, including humans (GTEx Analysis Release V7). We previously profiled Day 1 naïve and long-term memory-trained *crh-1/CREB* and wild-type animals to identify CREB’s basal and memory target genes, respectively [27]. CREB’s memory targets are highly enriched for genes with predicted neuronal expression (Fig 7A), and many of them are required for associative long-term memory [27]. Interestingly, memory training also induced the CREB-dependent transcription of many genes strongly predicted to be selectively expressed in the hypodermis and intestine (Fig 7A); how these non-neuronal genes might influence memory is unknown. By comparison, a large cluster of CREB’s basal targets in young adult animals is enriched for expression in the reproductive system, with the highest average expression of targets predicted in the hypodermis, muscle, and intestine, but not neurons (Fig 7B). CREB is involved in mammalian spermatogenesis and oocyte maturation [70,71], and CREB’s regulation of reproductive system expressed genes suggests that CREB may play a role in conserved pathways involved in reproduction and fertility. Thus, the prediction tool can help suggest new hypotheses for pathway functions from existing large, whole-worm transcriptional datasets.

#### Conclusions

*C*. *elegans’* simple architecture lends itself well to analysis of tissue-specific functions, and here we have exploited its simplicity to probe tissue-specific expression of adult animals. The isolation and RNA-seq analysis of *C*. *elegans* adults’ major somatic tissues has revealed that the transcriptomes of tissues differ from one another, and also from their counterparts in embryonic and larval stages, highlighting the importance of transcriptionally profiling specific developmental stages and cells relevant for the corresponding biology. These data provide the framework to explore the genetic basis of many phenotypes whose transcriptional underpinnings were not well represented with available larval data. The most striking example of this is the hypodermis, a tissue that is critically involved in development-specific larval molting processes, but in adults also becomes associated with metabolism, as shown here. These differences are likely to reveal the underlying basis of the hypodermis’ role in the regulation of reproductive aging, immunity, and longevity. The identification and prediction of insulin/FOXO target gene expression in non-intestine tissues, including hypodermis and neurons, lays the groundwork for understanding how lifespan signals are regulated within and across tissues. Moreover, the transcriptional conservation of the worm hypodermis and the human liver, in addition to other worm/human tissue similarities demonstrated here, provides new opportunities to model human diseases using genetic tools and resources of *C*. *elegans*.

We have provided tissue-specific transcriptional information, enrichment lists, GO analyses, and a new gene expression/cell type prediction tool for general usage. Our tool expands our knowledge of tissue- and cell-type specific expression to genome-wide coverage of 76 tissues and cell types, and is readily extendable as gene expression data grow. Our identification of tissue-specific isoform expression will enrich our understanding of differential uses for specific variants in different cellular contexts, and our prediction tool can be used to generate new hypotheses for gene function in specific cells. Together, these methods and these gene expression data will enhance the ability to understand adult biological function.

## Materials and methods

### Strains and worm cultivation

OH441: otIs45[P*unc-119*::*GFP*], CQ163: wqEx34[*Pmyo-3*::*mCherry*], CQ171: [*Py37a1b*.*5*::*GFP*], BC12890: [*dpy-5(e907)I; sIs11337*(*rCesY37A1B*.*5*::*GFP* + pCeh361), SJ4144: *zcIs18* (*Pges-1*::*GFP*), CQ236: *Pcrh-1g*::*GFP* + *Pmyo-2*::*mcherry*. Worm strains were maintained at 20°C on HGM plates using *E*. *coli* OP50. Strains were synchronized using hypochlorite treatment prior cell isolation and grown to day 1 of adulthood on HGM plates with *E*. *coli* OP50.

### Adult cell isolation

Synchronized day 1 adult worms with GFP-labeled neurons, muscle, hypodermis, and intestine (*Punc119*::*GFP*, *Pmyo-3*::*mCherry*, *pY37A1B*.*5*::*GFP*, and *Pges-1*::*GFP*) were prepared for cell isolation, as previously described [2].

### FACS isolation of dissociated cells

Cells were briefly subjected to SDS-DTT treatment, proteolysis, mechanical disruption, cell filtering, FACS, RNA amplification, library preparation, and single-end (140 nt) Illumina sequencing, as previously described [2]. Neuron cell suspensions were passed over a 5 μm syringe filter (Millipore). Muscle and hypodermal samples were gently passed over a 20 mm nylon filter (Sefar Filtration). Intestinal cells were passed through a 35 mm filter and by spinning at 500 x g for 30s in a tabletop centrifuge. The filtered cells were diluted in PBS/2% FBS and sorted using a either a FACSVantage SE w/ DiVa (BD Biosciences; 488nm excitation, 530/30nm bandpass filter for GFP detection) or a Bio-Rad S3 Cell Sorter (Bio-Rad; 488nm excitation). Gates for detection were set by comparison to N2 cell suspensions prepared on the same day from a population of worms synchronized alongside the experimental samples. Positive fluorescent events were sorted directly into Eppendorf tubes containing Trizol LS for subsequent RNA extraction. For each sample, approximately 50,000–250,000 GFP or mCherry positive events were collected, yielding 5–25 ng total RNA. Both sorters were used for each tissue, and the type of sorter did not affect the distribution of samples by multidimensional scaling analysis (Fig 1B), suggesting that the sorter did not contribute to the variability between samples of a given tissue.

### RNA isolation, amplification, library preparation, and sequencing

RNA was isolated from FACS-sorted samples as previously described [2]. Briefly, RNA was extracted using standard Trizol/ chloroform/ isopropanol method, DNase digested, and cleaned using Qiagen RNEasy Minelute columns. Agilent Bioanalyzer RNA Pico chips were used to assess quality and quantity of isolated RNA. 10 to 100 ng of the isolated quality assessed RNA was then amplified using the Nugen Ovation RNAseq v2 kit, as per manufacturer suggested practices. The resultant cDNA was then sheared to an average size of ~200 bp using Covaris E220. Libraries were prepared using either Nugen Encore NGS Library System or the Illumina TruSeq DNA Sample Prep, 1 μg of amplified cDNA was used as input. RNA from a subset of samples was amplified using the SMARTer Stranded Total RNA kit-pico input mammalian, as per manufacturer suggested practices. No differences were observed between the two methods, and samples amplified by different methods clustered well (Fig 1B). The resultant sequencing libraries were then submitted for sequencing on the Illumina HiSeq 2000 platform. 35–200 million reads (average of 107,674,388 reads) were obtained for each sample and mapped to the *C*. *elegans* genome. Sequences are deposited at NCBI BioProject PRJNA400796.

### RNA-seq data analysis

FASTQC was used to inspect the quality scores of the raw sequence data, and to look for biases. The first 10 bases of each read were trimmed before adapter trimming, followed by trimming the 3’ end of reads to remove the universal Illumina adapter and imposing a base quality score cutoff of 28 using Cutadapt v1.6 The trimmed reads were mapped to the *C*. *elegans* genome (Ensembl 84/WormBase 235) using STAR [72] with Ensembl gene model annotations (using default parameters). Count matrices were generated for the number of reads overlapping with the gene body of protein coding genes using featureCounts [73]. The per-gene count matrices were subjected to an expression detection threshold of 1 count per million reads per gene in at least 5 samples. EdgeR [74] was used for differential expression analysis and the multidimensional scaling (MDS) analysis. MDS is a method that aims to visualize proximity data in such a way that best preserves between-sample distances and is a commonly used technique (similar to PCA) to transform higher-dimension dissimilarity data into a two-dimensional plot. Here, we used the log-fold-change of expression between genes to compute distances. Genes at FDR = 0.05 were considered significantly differentially expressed. DEXSeq [75] was used for differential exon usage (splicing) analysis.

### Downsampling analysis

Count matrices of the aligned sequencing data were down-sampled using subSeq [15]. Reads were down-sampled at proportions using 10^x, starting at x = -5 and increasing at 0.25 increments to 0. The down-sampled count matrices were used to assess stability of number of expressed genes detected at multiple depths (S1A Fig). Because of minimum library sizes for tractable differential exon usage analysis, reads with down-sampled proportions using 10^x, from x = -2, increasing at 0.25 increments to 0 were used for assessment of power in detecting differential splicing (S1B Fig).

### Gene ontology analysis

Hypergeometric tests of Gene Ontology terms were performed on tissue-enriched gene lists; GO terms reported are a significance of *q-value* < 0.05 unless otherwise noted. REVIGO was used to cluster and plot GO terms with q-value < 0.05.

### Motif analysis

RSAtools [76] was used to identify the -1000 to -1 promoter regions of the tissue enriched genes and perform motif analysis. Matrices identified from RSAtools were analyzed using footprintDB [77] to identify transcription factors predicted to bind to similar DNA motifs. Alternatively, motifs were analyzed using gProfiler [78].

### Oil Red O staining and analysis

Hypodermal genes appearing in metabolic GO terms were selected from the top of the tissue-enriched list (*aldo-2*, *gpd-2*, *sams-1*, *cth-2*, *pmt-1*, *idh-1*, and *fat-2*) or the expressed list (*far-2* and *gpd-3*) and knocked down using RNAi and compared to a vector (L4440) control. On day 1 of adulthood, all worms were stained in Oil Red O for 6–24 hours and then imaged using a Nikon Eclipse Ti microscope at 20x magnification [79]. Images were analyzed for mean intensity in fat objects using CellProfiler [80]. Additional genes from the hypodermal unique list were also selected and tested for fat (Oil Red O) levels.

### Worm-human tissue comparison

Human orthologs [30] of genes in our tissue-enriched gene lists were compared with curated tissue-specific gene annotations from the Human Protein Reference Database [31] for significant overlap (hypergeometric test).

### Identification of ‘tissue-enriched’ and ‘unique’ tissue-specific genes

‘Tissue-enriched’ genes are highly enriched relative to all other tissues, defined as genes that are highly expressed (log_RPKM_ > 5) and significantly differentially expressed relative to the average expression across all of the other tissues (FDR ≤ 0.05, log_FC_ > 2; S8 Table).

‘Unique’ tissue-specific genes are strongly expressed (log_RPKM_ > 5) and significantly differentially expressed in comparison to the expression of *each* of the three other tissues (FDR ≤ 0.05, log_FC_ > 2 for each comparison; S9 Table, S1E Fig).

### IIS/FOXO target expression in adult tissues

The expression level (expressed defined as log(rpkm) >2) for previously published IIS/FOXO targets (Tepper et al., 2013, cut-off 5% FDR) were identified for each tissue. Tissue overlaps were graphed in Venn diagrams using the Venn diagram package in R.

### Expression data compendium assembly

To construct these models, we needed a large data compendium and high quality examples of tissue expression. We assembled 273 *C*. *elegans* expression datasets (comprised of both adult and developmental expression data), representing 4,372 microarray and RNA-seq samples, including our tissue-ome library. All other datasets were downloaded from the Gene Expression Omnibus (GEO) data repository, ArrayExpress Archive of Functional Expression Data, and WormBase. Samples from each dataset were processed together (duplicate samples were collapsed, background correction and missing value imputation were executed when appropriate). Within each dataset, gene expression values were normalized to the standard normal distribution. All datasets were then concatenated, and genes that were absent in only a subset of datasets received values of 0 (in datasets in which they were absent). The predictions that were used to analyze the tissue-ome dataset were generated using a data compendium that excluded the tissue-ome library.

### Tissue expression gold standard construction

Gene annotations to tissues and cell types were obtained from curated anatomy associations from WormBase [81] (WS264) and other small-scale expression analyses as curated by Chikina et al. (2009). Only annotations based on smaller scale experiments (e.g., single-gene GFP, *in situ* experiments) were considered for the gold standard, excluding annotations derived from SAGE, microarray, RNA-seq, etc. Annotations from both adult and developing worm were considered. Annotations were mapped and propagated (up to each of its ancestor terms, e.g., a gene directly annotated to dopaminergic neuron would thus be propagated up to ancestor terms such as neuron and nervous system and included in the corresponding gold standards) based on the WormBase *C*. *elegans* Cell and Anatomy Ontology, where a stringent cutoff was used for which tissues and cell types were retained (>50 direct annotations and >150 propagated annotations).

We defined a “tissue-slim” based on system-level anatomy terms in the WormBase anatomy ontology (immediate children of “organ system” and “sex specific entity,” under “functional system”). The nine resulting terms are: alimentary system, coelomic system, epithelial system, excretory secretory system, hermaphrodite-specific, male-specific, muscular system, nervous system, and reproductive system. For each of the 76 tissues that were retained, a tissue-gene expression gold standard was constructed in which genes annotated (directly or through propagation, i.e., the gene has been associated with either the particular tissue or a part of that tissue in a smaller scale experiment) to the tissue were considered as positive examples. Genes that were annotated to other tissues, but not in the same tissue system, were considered negative examples. Thus, genes were assigned as positive or negative examples of tissue expression while taking into account the tissue hierarchy represented in the Cell and Anatomy Ontology.

### An interactive web interface to explore tissue-gene expression models

Our tissue-gene expression predictions and similarity profiles have all been made accessible at a dynamic, interactive website, http://worm.princeton.edu. From this interface, users can explore the predicted expression patterns of their gene(s) of interest. To facilitate this exploration, we have designed an interactive heatmap visualization that allows users to view hierarchically clustered expression patterns or sort by any gene or tissue model of interest. In addition, we also provide suggestions of genes with similar tissue expression profiles, which users can immediately visualize alongside their original query. All predictions and visualizations are available for direct download.

### Prediction and evaluation of tissue-gene expression profile and similarity

For each of the 76 tissues and cell types, we used the expression data compendium and corresponding gold standard as input into a linear support vector machine (SVM) to make predictions for every gene represented in our data. Specifically, given the vector of gene expression data (***x***_***i***_) and training label (*y*_*i*_:{-1,1}) for gene *i*, hyperplanes described by ***w*** and *b*, and constant *c*, the SVM’s objective function is:

$$\begin{array}{l} \underset{w,\xi }{\text{min}}\frac{1}{2}w^{T}w+c\sum_{i}\xi_{i},\;\text{subject}\;\text{to}\;\text{the}\;\text{constraints}:\\ y_{i}\left(w\cdot x_{i}-b\right)\geq 1-\;\xi_{i},\;\xi_{i}\geq 0. \end{array}$$

SVM parameters were optimized for precision at 10% recall under 5-fold cross validation. Resulting SVM scores were normalized to the standard normal distribution for any comparisons across tissues. Feature weights for each of the tissue SVM models were also retained for ranking and analysis of samples.

## Supporting information

S1 Fig Analysis of RNA-seq datasets. A) SubSeq analysis calculating number of expressed genes found per tissue at different sequencing depths. The identification of expressed genes stabilizes at approximately 10% of the final read depth. B) Heatmap showing read coverage profiles over gene body to evaluate whether coverage is uniform (versus potential 5’ or 3’ bias). C) SubSeq analysis calculating number of significant differentially expressed exons that would be identified at different sequencing depths. The identification of differential exon usage begins to saturate at the final read depth, demonstrating the necessity for deep sequencing.
(PDF)

S2 Fig Oil Red O staining of worms treated with RNAi targeting hypodermal genes. A) Comparison of tissue-enriched and unique genes. The unique genes comprise a subset of the tissue-enriched gene set. B) Spearman correlation of tissue-enriched gene expression in each sample. C) Percentage difference of mean intensity of Oil Red O staining relative to vector control. *aldo-2*, *far-2*, *gpd-3*, and *sams-1* RNAi-treated animals had significantly more fat content (* p-value < 0.05, **** p-value < 0.0001 by one-way ANOVA), compared to vector control. D) Representative 20x images of Day 1 RNAi-treated animals after 6 hours of Oil Red O staining. No significant differences in worm size were observed.
(PDF)

S3 Fig Computational evaluations of nervous and muscular system models. Precision-recall curves showing accuracy of predictions for nervous system (A) and muscular system (B) tissues and cell types. Dotted line indicates genomic background (i.e., the expected precision if genes were randomly chosen). Precision-recall curves show the tradeoff between precision and recall for different thresholds, where high precision corresponds to a low false positive rate. Because every gene in the genome is given a prediction, an examination of the complete list results in a recall of 1.
(PDF)

S4 Fig Computational evaluations of alimentary, epithelial coelomic, and excretory system models. Precision-recall curves showing accuracy of predictions for alimentary system (A), epithelial system (B), coelomic system (C), and excretory system (D) tissues and cell types. Dotted line indicates genomic background.
(PDF)

S5 Fig Computational evaluations of reproductive system, hermaphrodite-specific, and male-specific tissue models. Precision-recall curves showing accuracy of predictions for reproductive system (A), hermaphrodite-specific (B), and male-specific (C), tissues and cell types. Dotted line indicates genomic background.
(PDF)

S6 Fig Individual gene predictions. A) Predicted expression of *daf-2*, *daf-16*, *pqm-1*, and *crh-1*. B) Insulin genes were analyzed for predicted tissue expression (red = highest predicted expression, blue = lowest predicted expression). Gold standard annotations are represented with the highest score possible. Tissues are listed by average gene expression score.
(PDF)

S7 Fig Predictions and curated annotations for insulin genes. A) Predictions only are shown for the insulin gene family (red = highest predicted expression, blue = lowest predicted expression). B) Wormbase curated annotations are shown (black = expressed, grey = unannotated).
(PDF)

S8 Fig Tissue predictions for Class I and Class II IIS genes. The top 50 Class I (A) and Class II genes (B) [23] were analyzed for predicted tissue expression (red = highest predicted expression, blue = lowest predicted expression). Gold standard annotations are represented with the highest score possible. Tissues are listed by average gene expression score.
(PDF)

S9 Fig Tissue predictions for TGF-β-regulated genes. A) Upregulated genes from microarray analysis of wild type (N2) vs *sma-2* oocytes [57] were analyzed for predicted tissue expression. B) Whole worm upregulated genes from microarray analysis of wild type (N2) vs *sma-2* L4 animals [57] were analyzed for predicted tissue expression.
(PDF)

S1 Table Expressed genes. Genes for each tissue are listed based upon an expression detection threshold of 1 count per million reads per gene in at least 5 samples.
(XLSX)

S2 Table Ubiquitous and tissue-enriched GO terms. Significant gene ontology (GO) terms are listed for genes with ubiquitous expression (S1 Table) or for tissue-enriched genes (S8 Table).
(XLSX)

S3 Table Stage-specific neuron GO terms. Stage-specific and stage-overlapping genes from the comparison of larval, embryonic [1] and adult neurons were used for GO analysis.
(XLSX)

S4 Table Stage-specific hypodermis GO terms. Stage-specific and stage-overlapping genes from the comparison of larval, embryonic [1] and adult hypodermis were used for GO analysis.
(XLSX)

S5 Table Stage-specific muscle GO terms. Stage-specific and stage-overlapping genes from the comparison of larval, embryonic [1]and adult muscle were used for GO analysis.
(XLSX)

S6 Table Stage-specific intestine GO terms. Stage-specific and stage-overlapping genes from the comparison of larval, embryonic [1] and adult intestine were used for GO analysis.
(XLSX)

S7 Table Gene subsets. Classes of genes, including transcription factors, forkhead transcription factors, secreted proteins, insulins, nuclear hormone receptors, and transmembrane proteins expressed in each tissue type are listed.
(XLSX)

S8 Table Tissue-enriched genes. ‘Tissue-enriched’ genes are highly enriched relative to all other tissues, defined as genes that are highly expressed (log_RPKM_ > 5) and significantly differentially expressed relative to the average expression across all of the other tissues (FDR ≤ 0.05, log_FC_ > 2). Transcription factors (wTF3.0[86]) are listed in red.
(XLSX)

S9 Table Unique genes. ‘Unique’ tissue-specific genes are strongly expressed (log_RPKM_ > 5) and significantly differentially expressed in comparison to the expression of *each* of the three other tissues (FDR ≤ 0.05, log_FC_ > 2 for each comparison). Transcription factors (wTF3.0[86]) are listed in red.
(XLSX)

S10 Table Tissue-enriched motif analysis. Matrices identified from RSAtools were analyzed using footprintDB. The 1kb promoter input from the tissue-enriched genes, RSAtools enriched motifs, and significant footprintDB binding factors are listed.
(XLSX)

S11 Table Comparison of worms and human tissues. Human orthologs of genes in our tissue-enriched gene lists were compared with curated tissue-specific gene annotations from the Human Protein Reference Database for significant overlap (hypergeometric test). Significant overlapping tissues, genes from each overlapping category, and GO terms of worm orthologs from each overlapping category are provided.
(XLSX)

S12 Table Tissue-enrichment of alternative splicing. DEXSeq was used for differential exon usage (splicing) analysis. Significant splicing events for each tissue are listed.
(TXT)

S13 Table IIS/FOXO genes in wild-type tissues. Adult tissue expression levels of the wild-type worm Class I and Class II IIS-FOXO genes from Tepper et al., 2013.
(XLSX)
