# Supplementary figures and images for: Transcriptome analysis of adult Caenorhabditis elegans cells reveals tissue-specific gene and isoform expression
Source: PLoS Genet. 2018 Aug 10;14(8):e1007559. doi: 10.1371/journal.pgen.1007559 (PMC6105014; doi:10.1371/journal.pgen.1007559)

A

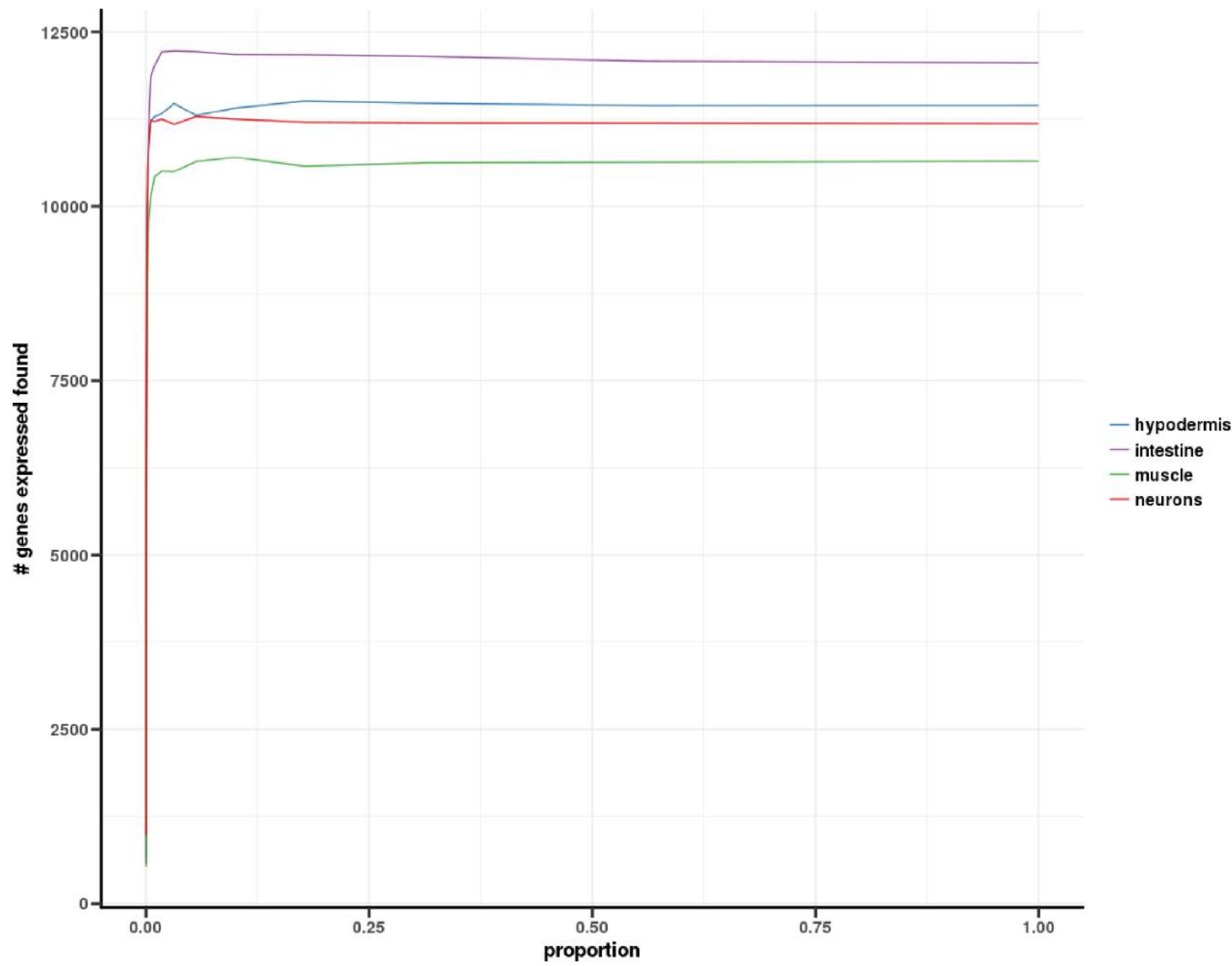

B

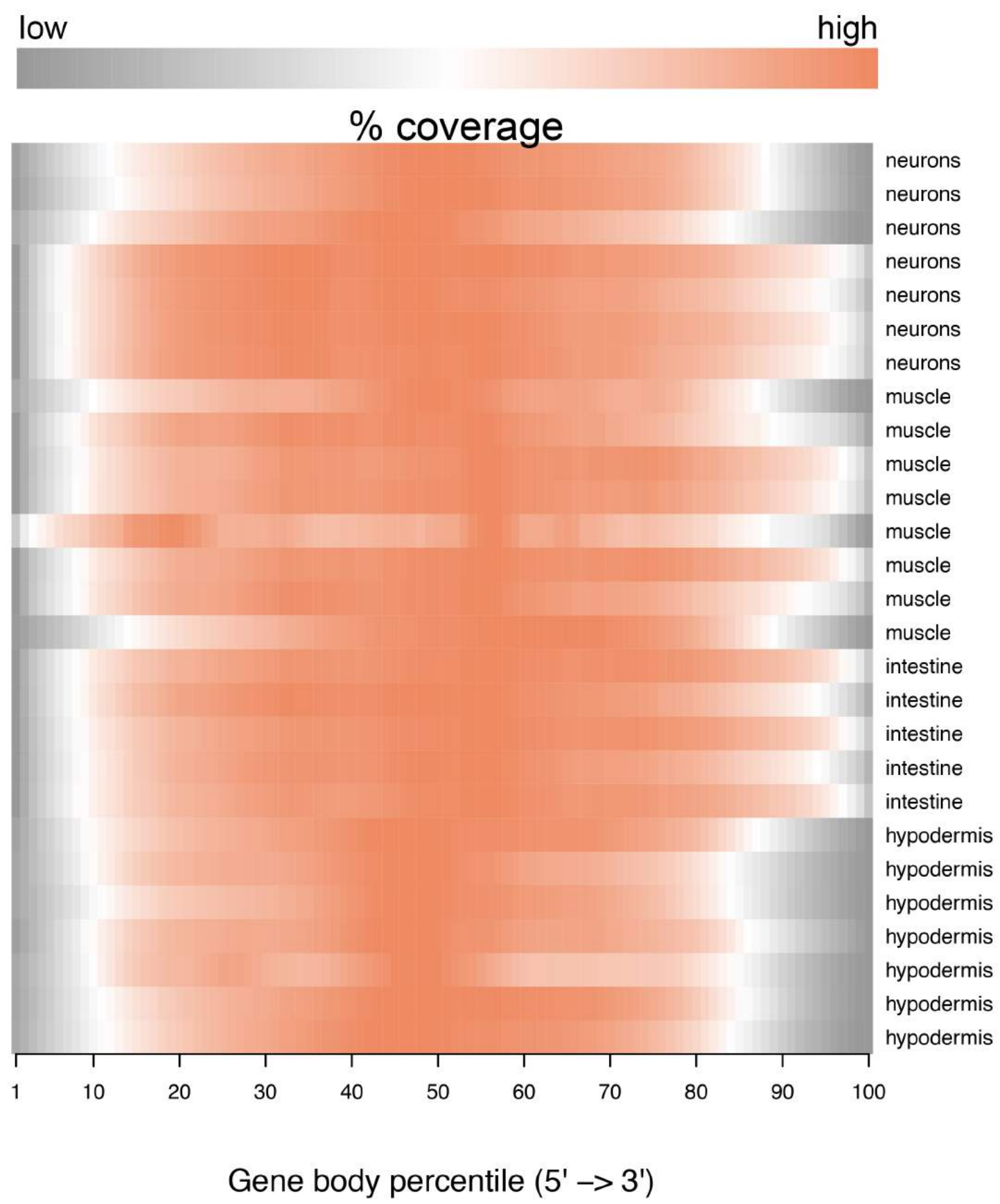

C

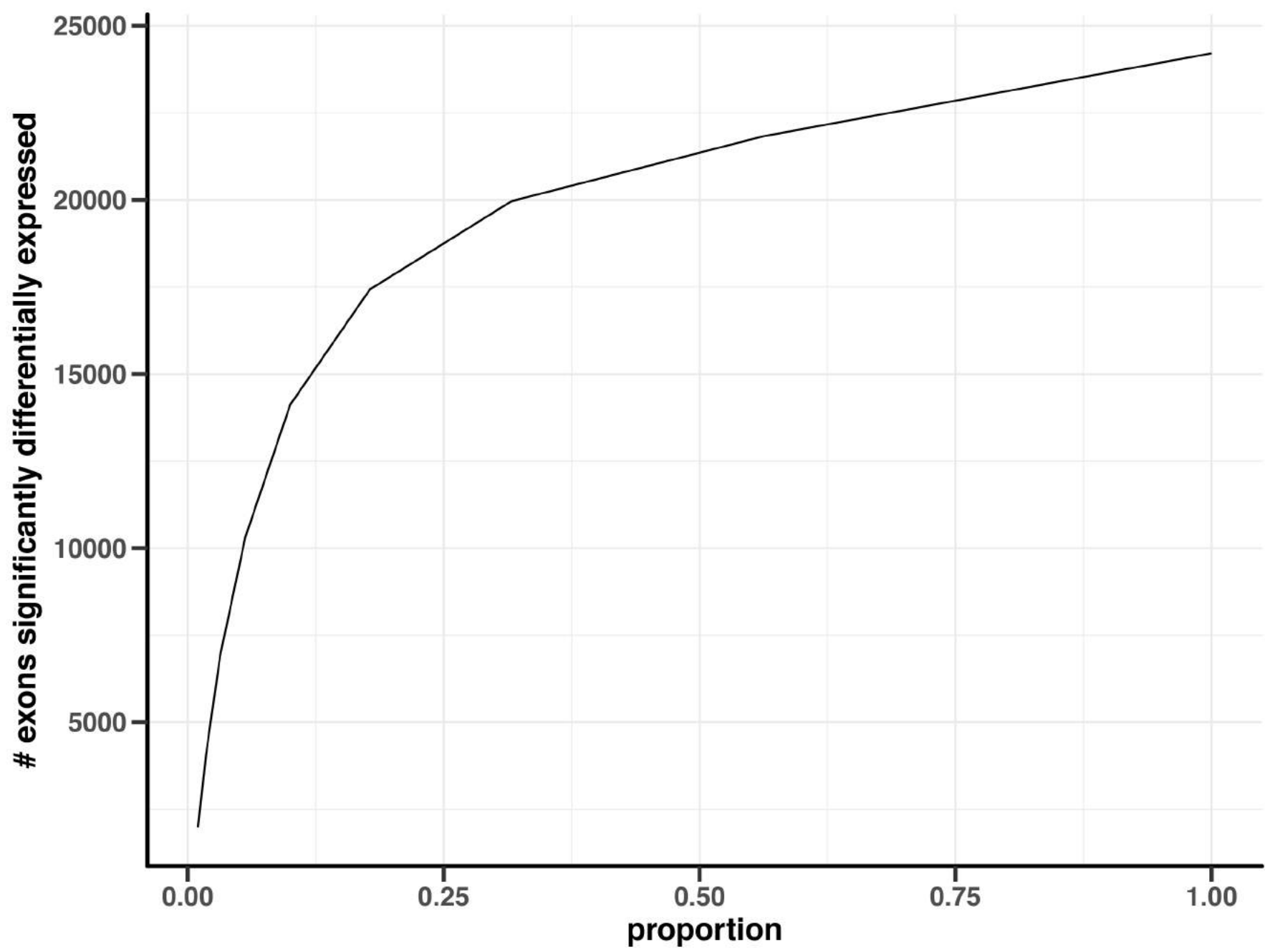

Supplemental Figure 1

Supplement: S1 Fig — A) SubSeq analysis calculating number of expressed genes found per tissue at different sequencing depths. The identification of expressed genes stabilizes at approximately 10% of the final read depth. B) Heatmap showing read coverage profiles over gene body to evaluate whether coverage is uniform (versus potential 5’ or 3’ bias). C) SubSeq analysis calculating number of significant differentially expressed exons that would be identified at different sequencing depths. The identification of differential exon usage begins to saturate at the final read depth, demonstrating the necessity for deep sequencing. (PDF) [file pgen.1007559.s001.pdf]

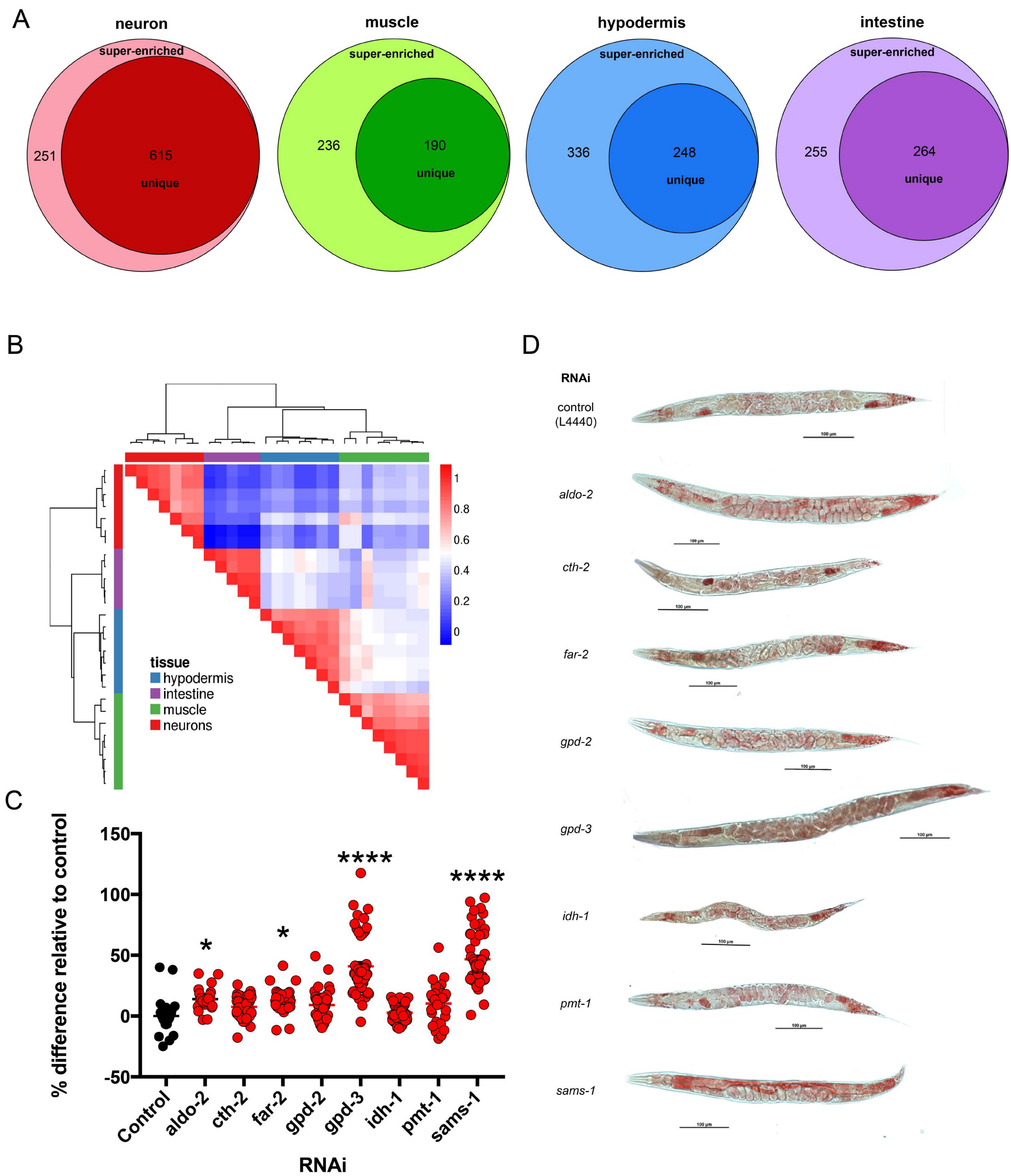

Supplemental Figure 2

Supplement: S2 Fig — A) Comparison of tissue-enriched and unique genes. The unique genes comprise a subset of the tissue-enriched gene set. B) Spearman correlation of tissue-enriched gene expression in each sample. C) Percentage difference of mean intensity of Oil Red O staining relative to vector control. aldo-2, far-2, gpd-3, and sams-1 RNAi-treated animals had significantly more fat content (* p-value < 0.05, **** p-value < 0.0001 by one-way ANOVA), compared to vector control. D) Representative 20x images of Day 1 RNAi-treated animals after 6 hours of Oil Red O staining. No significant differences in worm size were observed. (PDF) [file pgen.1007559.s002.pdf]

A

nervous system tissues

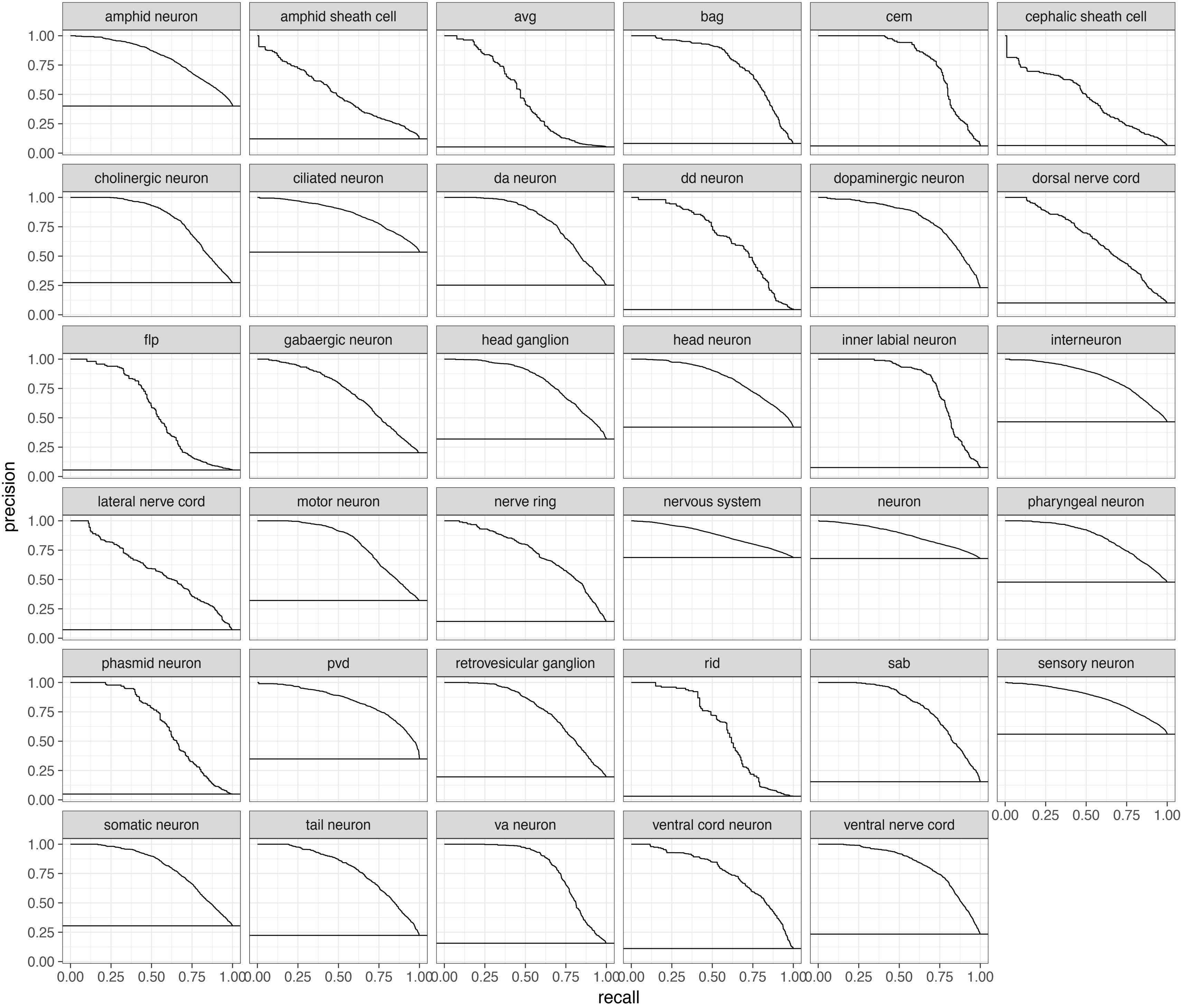

B

muscular system tissues

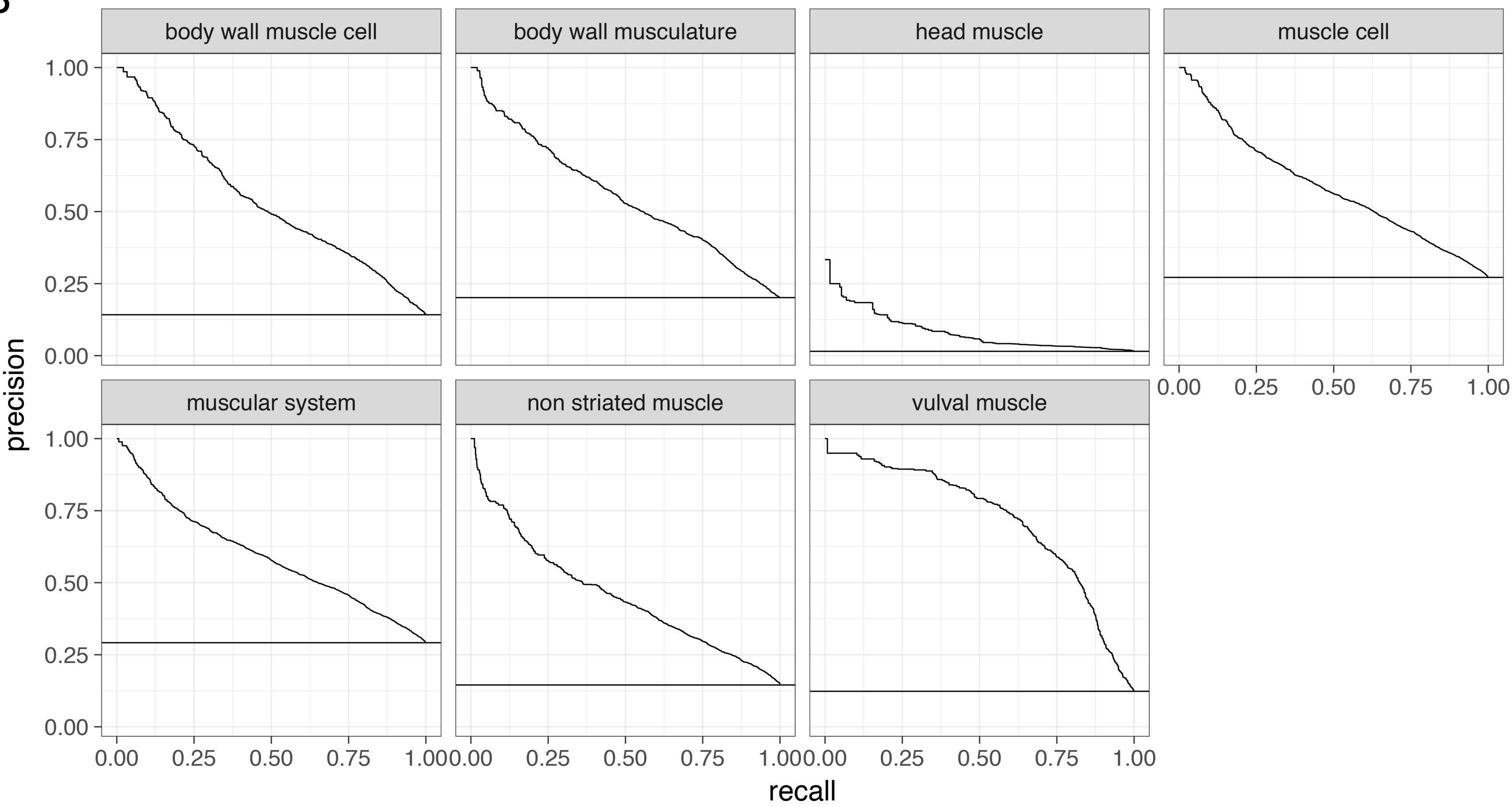

Supplemental Figure 3

Supplement: S3 Fig — Precision-recall curves showing accuracy of predictions for nervous system (A) and muscular system (B) tissues and cell types. Dotted line indicates genomic background (i.e., the expected precision if genes were randomly chosen). Precision-recall curves show the tradeoff between precision and recall for different thresholds, where high precision corresponds to a low false positive rate. Because every gene in the genome is given a prediction, an examination of the complete list results in a recall of 1. (PDF) [file pgen.1007559.s003.pdf]

A

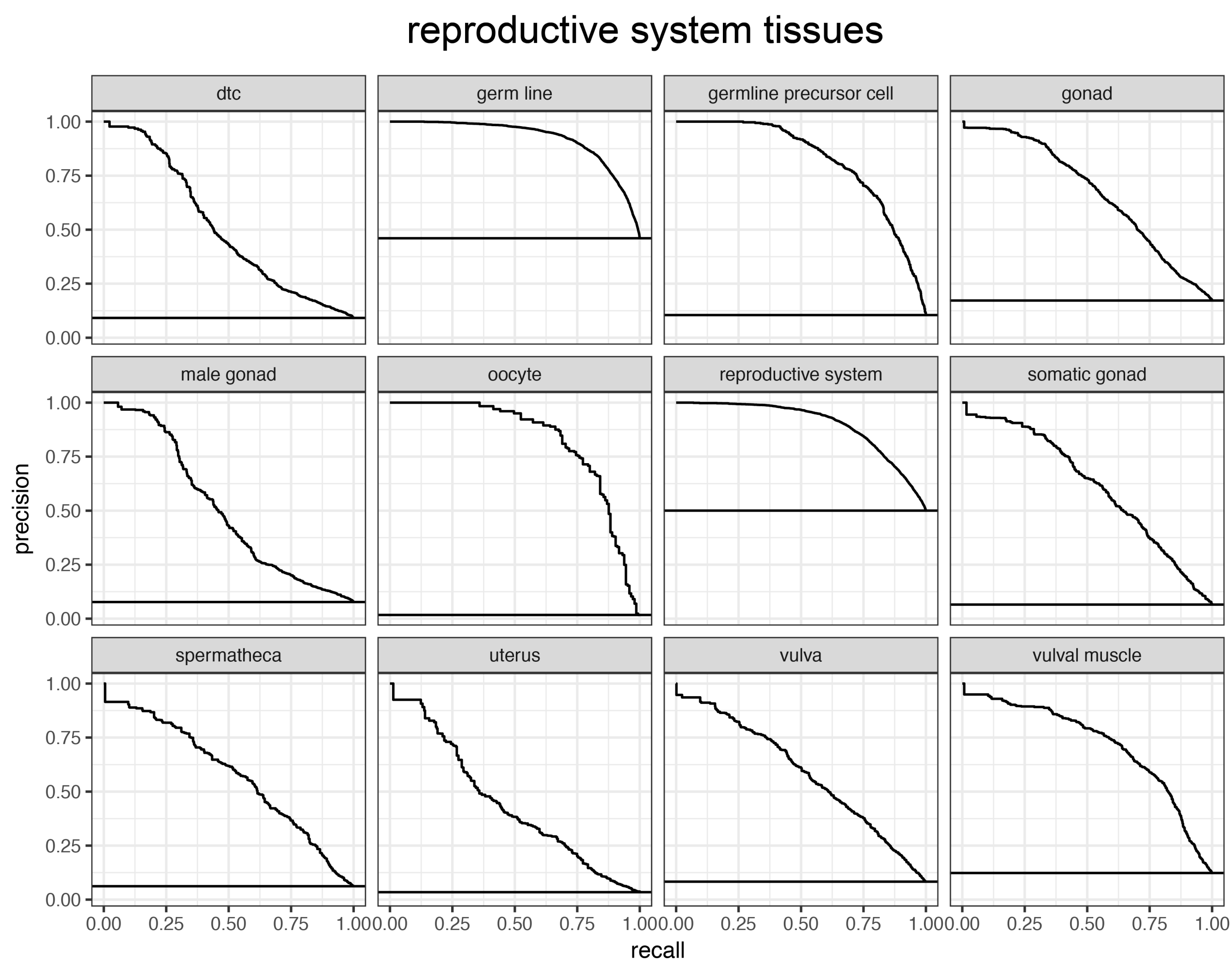

B

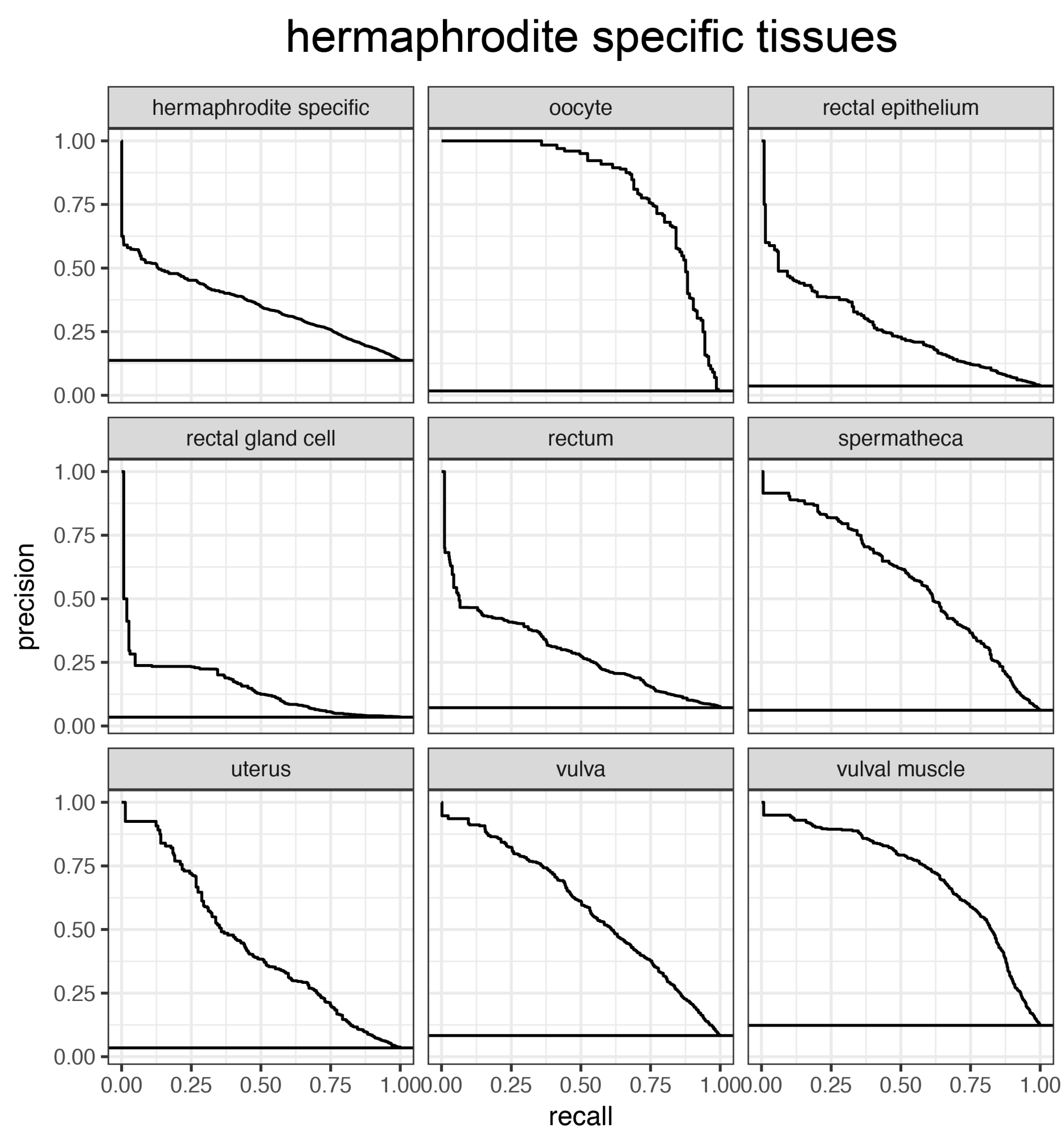

C

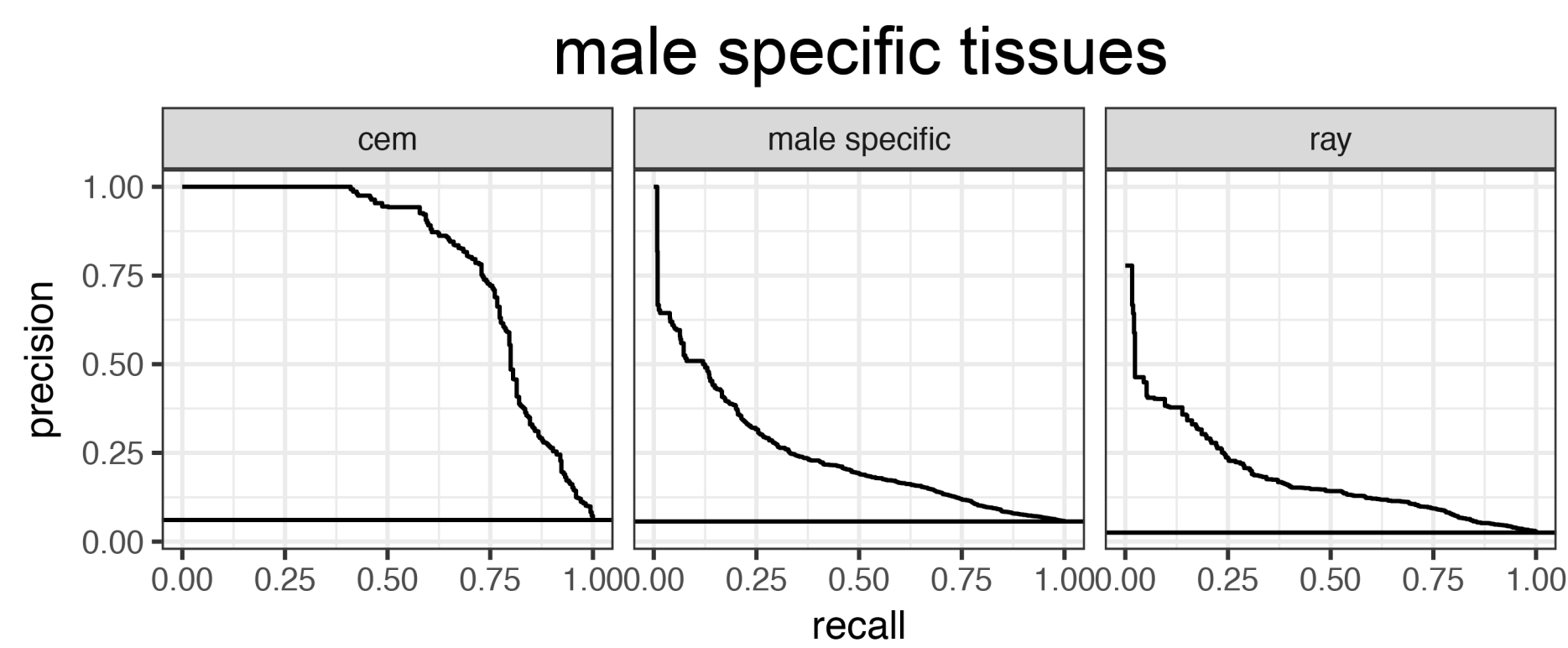

Supplement: S5 Fig — Precision-recall curves showing accuracy of predictions for reproductive system (A), hermaphrodite-specific (B), and male-specific (C), tissues and cell types. Dotted line indicates genomic background. (PDF) [file pgen.1007559.s005.pdf]

A

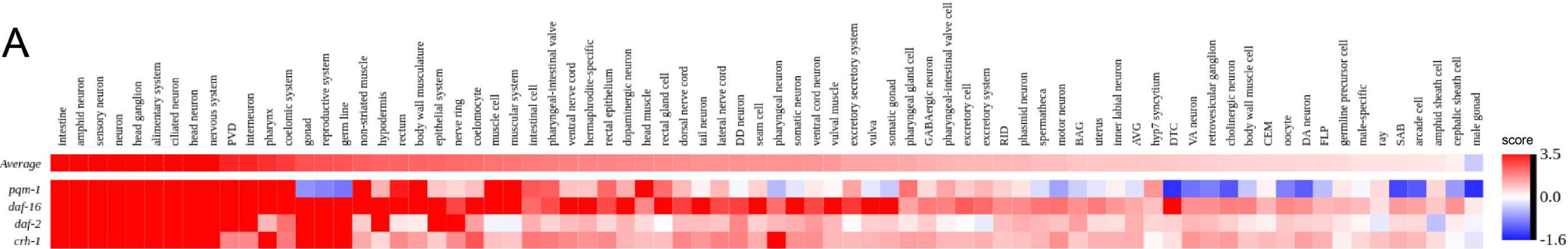

B

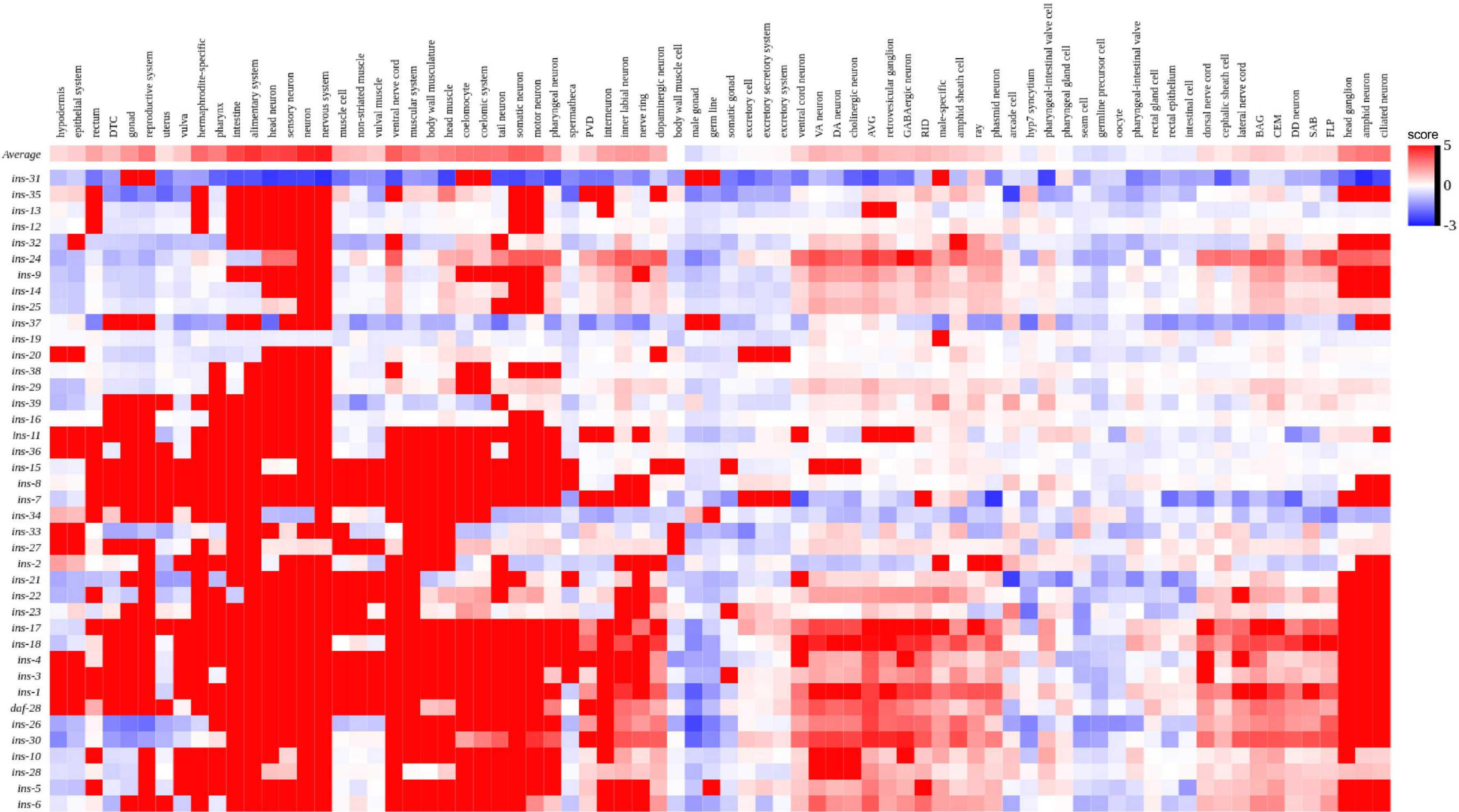

Supplemental Figure 6

Supplement: S6 Fig — A) Predicted expression of daf-2, daf-16, pqm-1, and crh-1. B) Insulin genes were analyzed for predicted tissue expression (red = highest predicted expression, blue = lowest predicted expression). Gold standard annotations are represented with the highest score possible. Tissues are listed by average gene expression score. (PDF) [file pgen.1007559.s006.pdf]

A

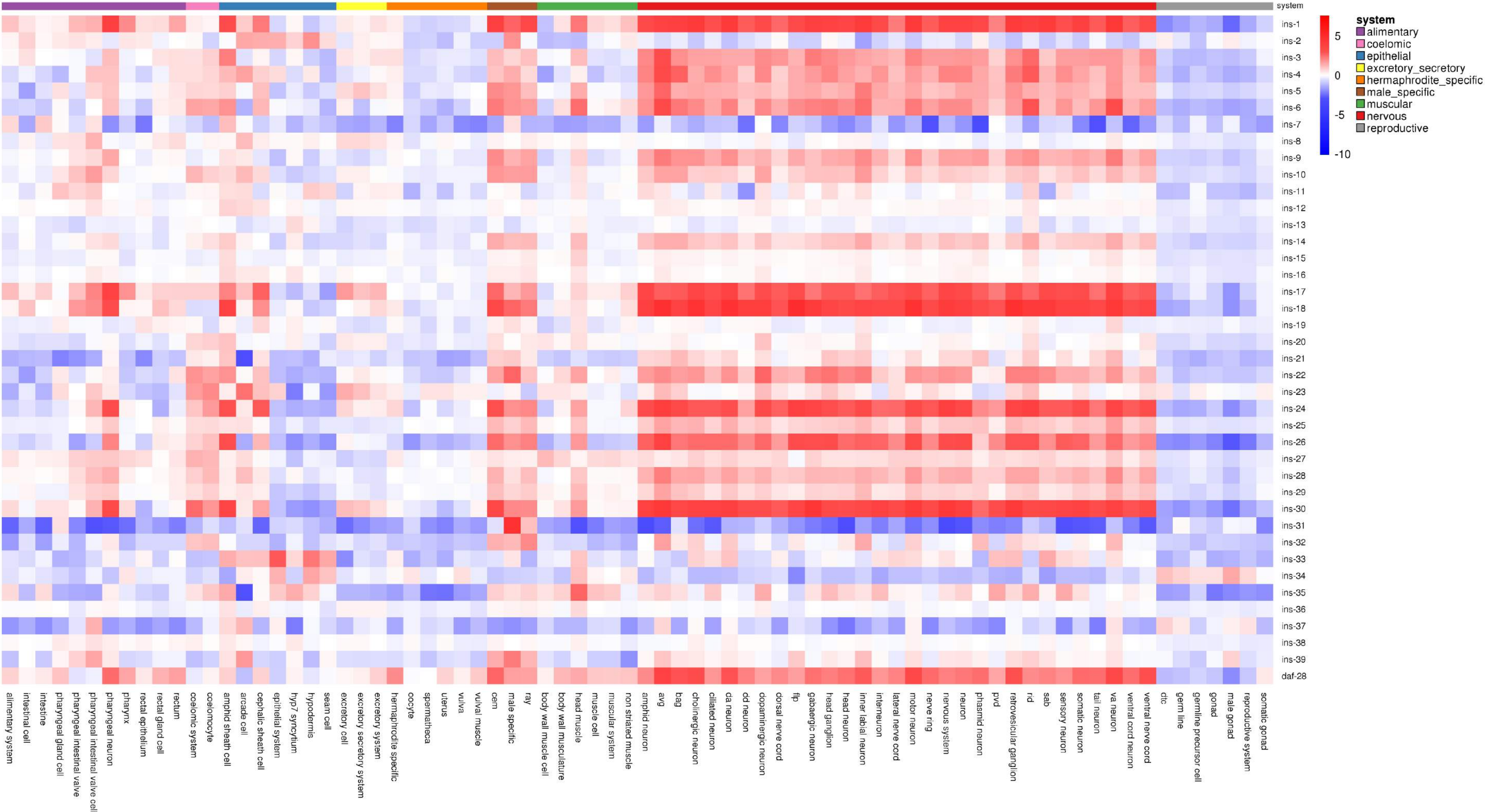

B

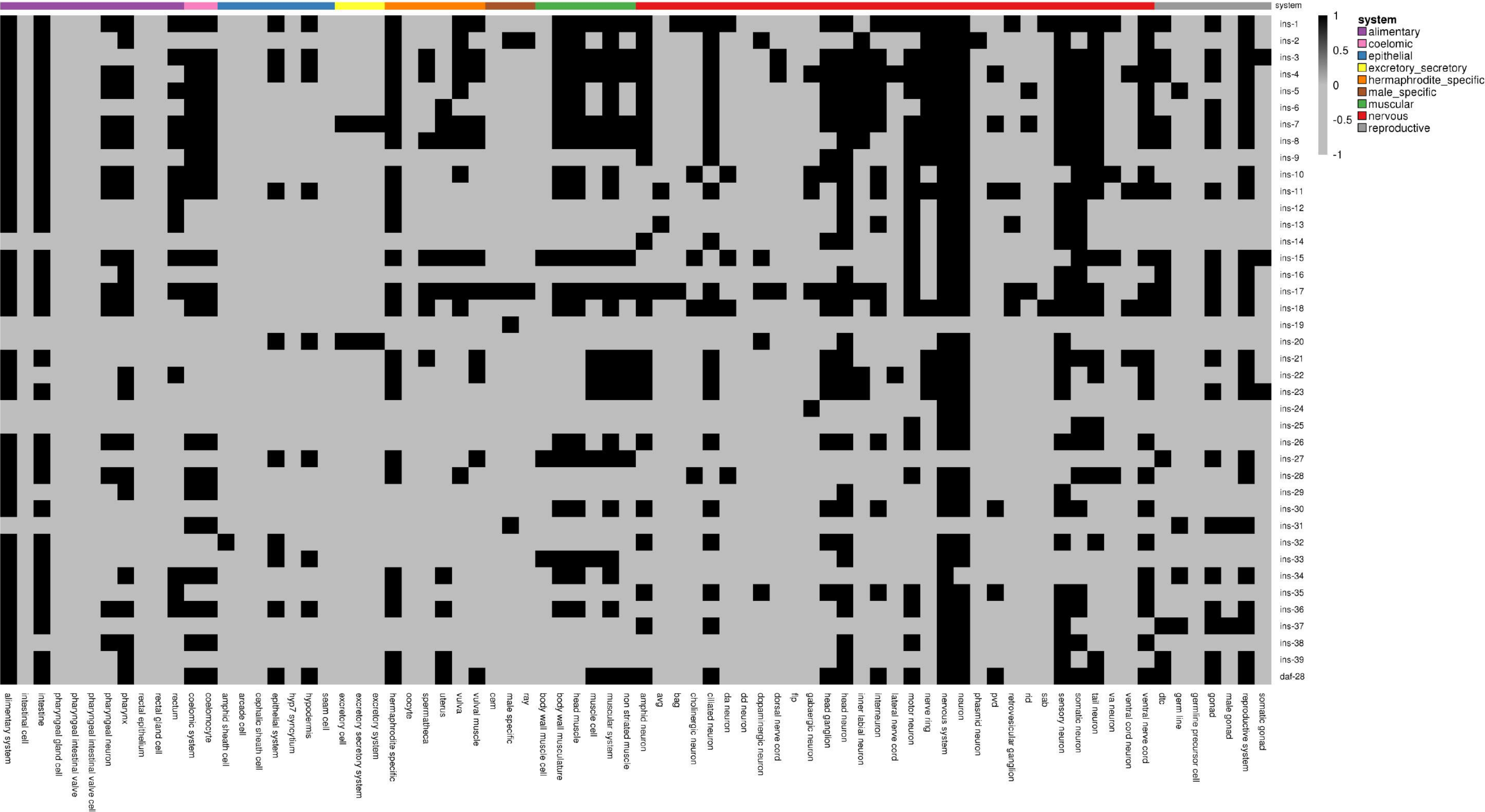

Supplemental Figure 7

Supplement: S7 Fig — A) Predictions only are shown for the insulin gene family (red = highest predicted expression, blue = lowest predicted expression). B) Wormbase curated annotations are shown (black = expressed, grey = unannotated). (PDF) [file pgen.1007559.s007.pdf]

A

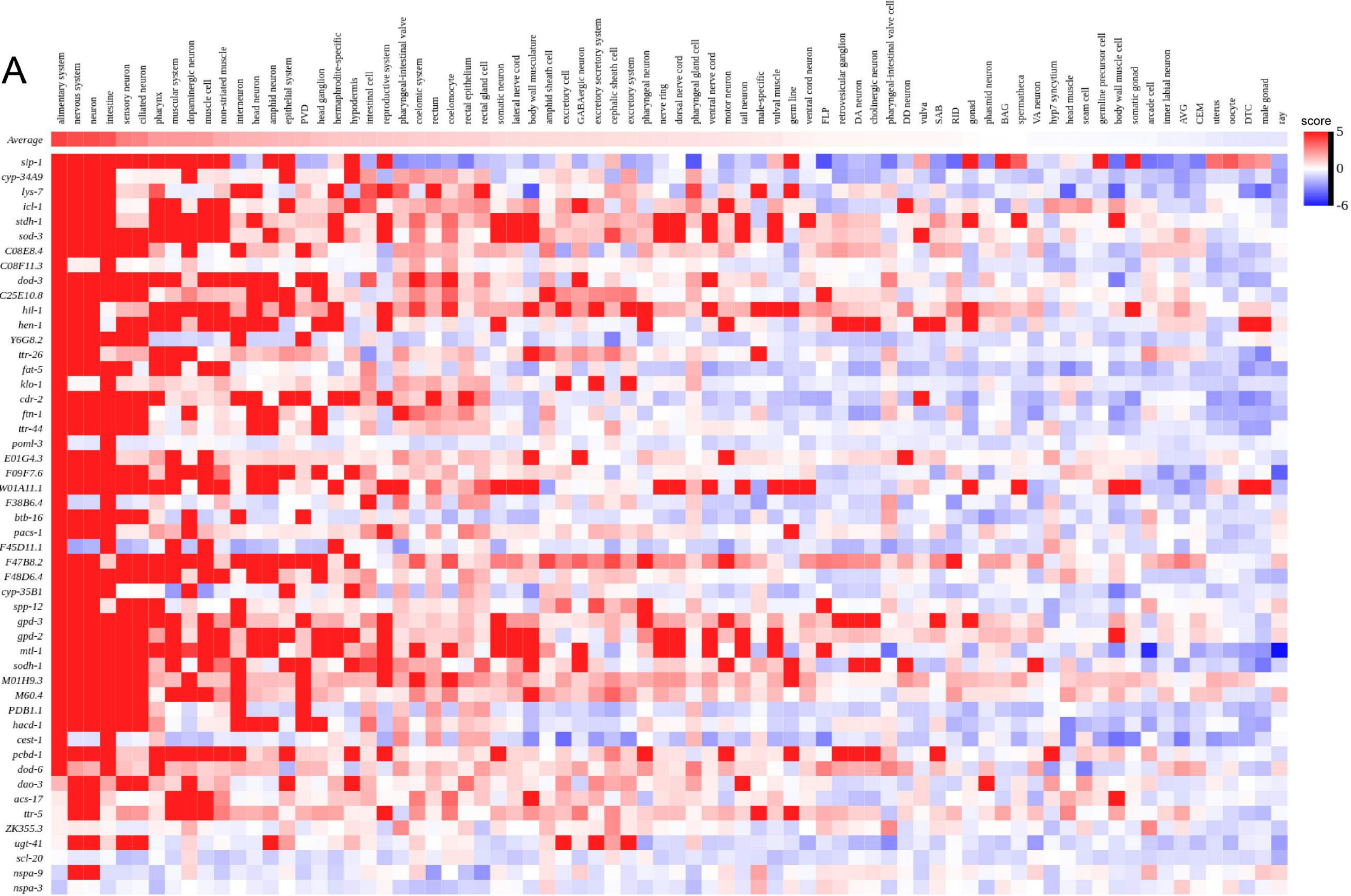

B

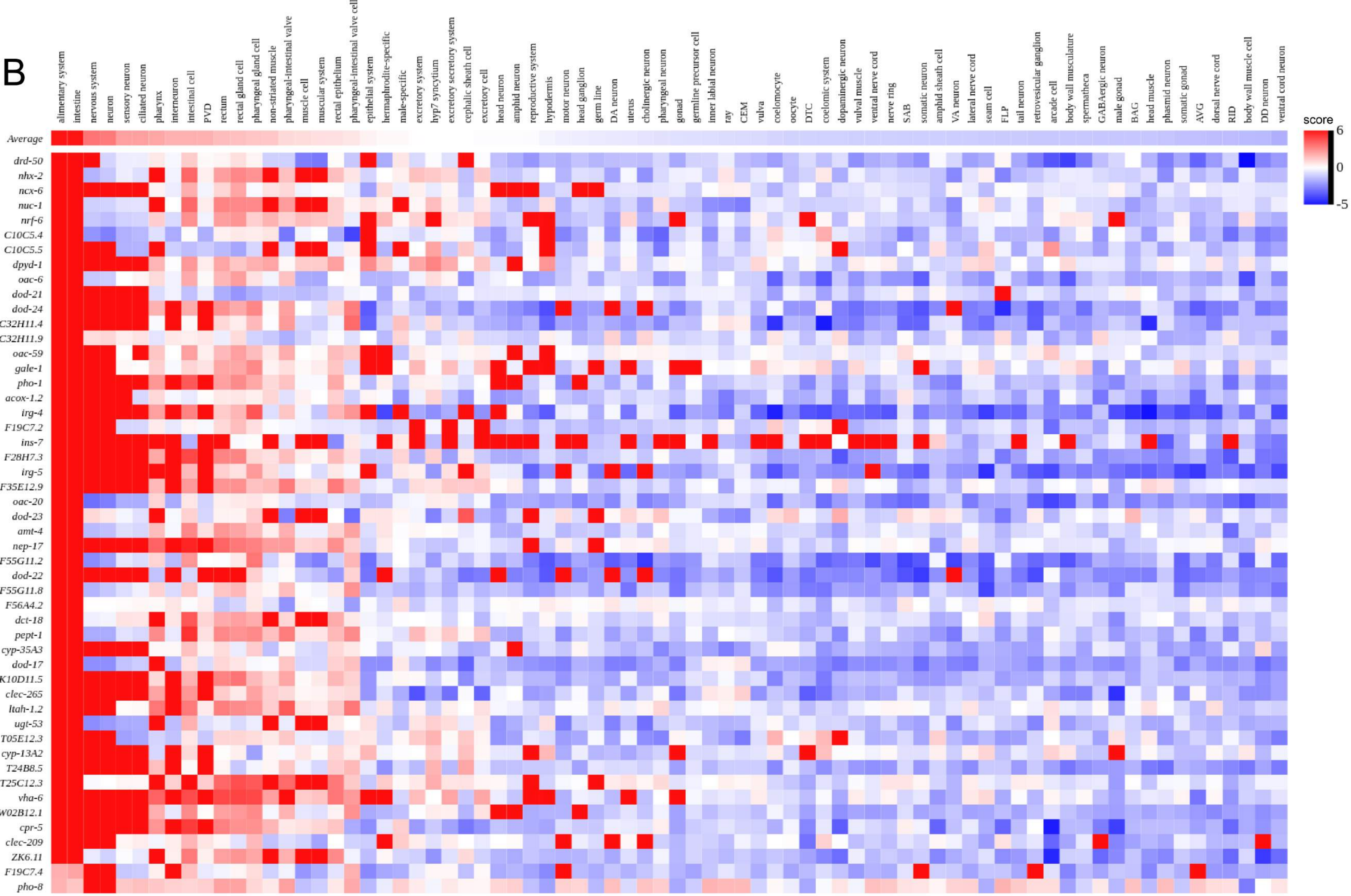

Supplemental Figure 8

Supplement: S8 Fig — The top 50 Class I (A) and Class II genes (B) [23] were analyzed for predicted tissue expression (red = highest predicted expression, blue = lowest predicted expression). Gold standard annotations are represented with the highest score possible. Tissues are listed by average gene expression score. (PDF) [file pgen.1007559.s008.pdf]

A

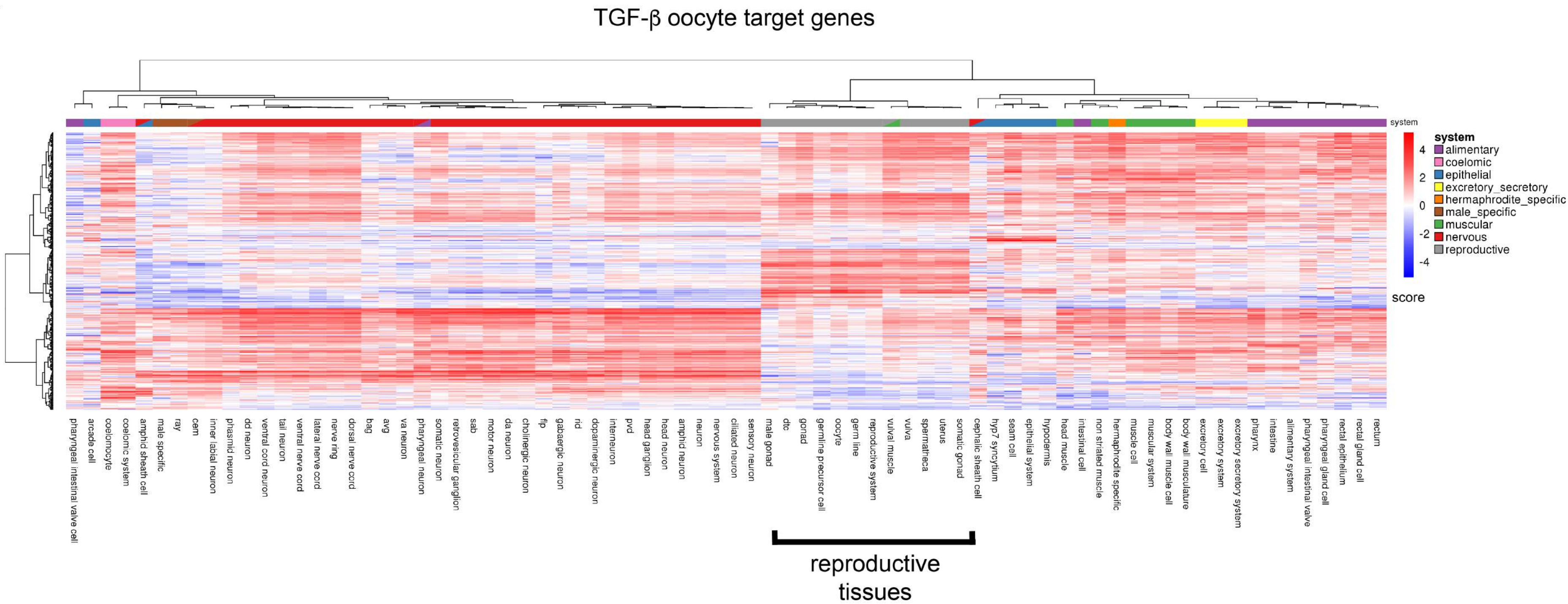

B

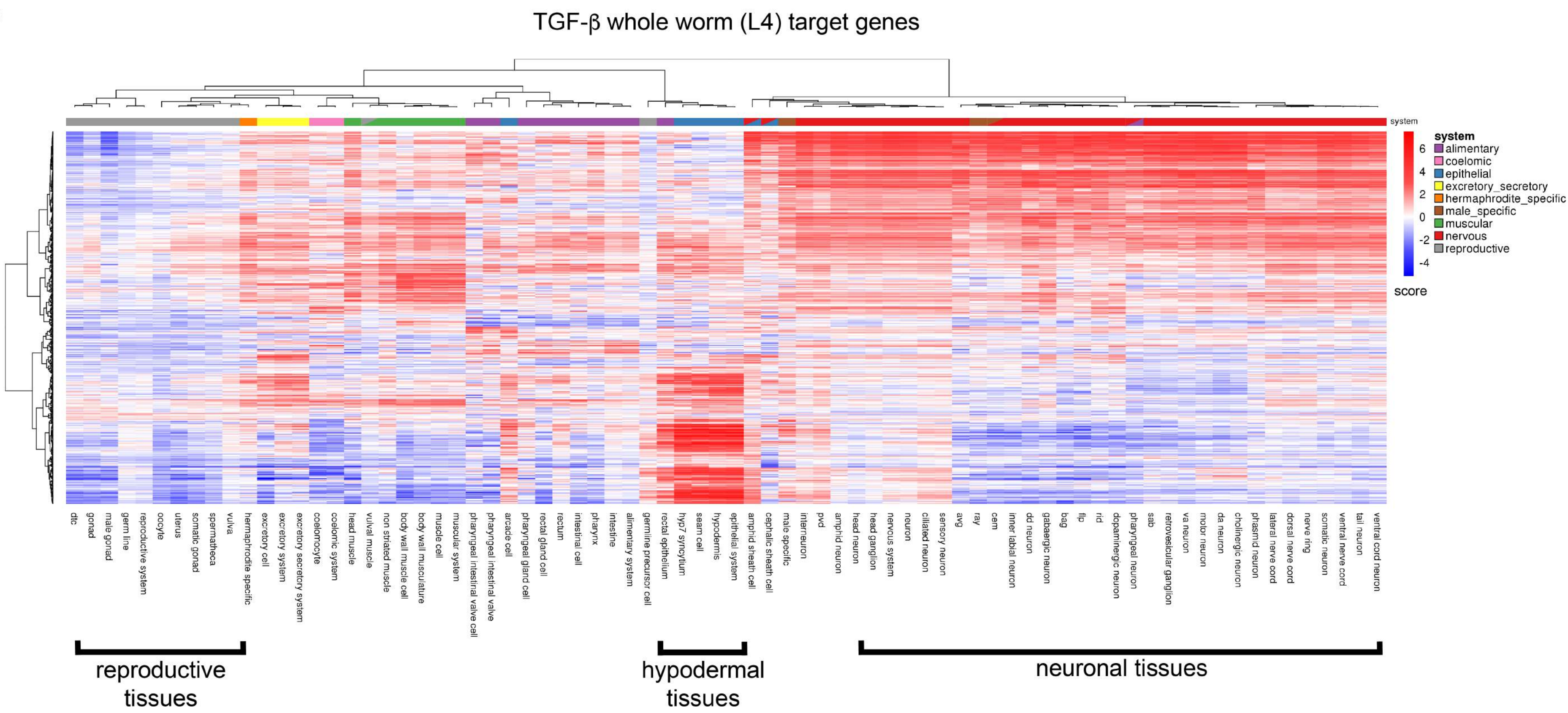

Supplemental Figure 9

Supplement: S9 Fig — A) Upregulated genes from microarray analysis of wild type (N2) vs sma-2 oocytes [57] were analyzed for predicted tissue expression. B) Whole worm upregulated genes from microarray analysis of wild type (N2) vs sma-2 L4 animals [57] were analyzed for predicted tissue expression. (PDF) [file pgen.1007559.s009.pdf]
