# Supplementary material for: Transcriptome analysis of adult Caenorhabditis elegans cells reveals tissue-specific gene and isoform expression
Source: PLoS Genet. 2018 Aug 10;14(8):e1007559. doi: 10.1371/journal.pgen.1007559 (PMC6105014; doi:10.1371/journal.pgen.1007559)
Supplement: S4 Fig — Precision-recall curves showing accuracy of predictions for alimentary system (A), epithelial system (B), coelomic system (C), and excretory system (D) tissues and cell types. Dotted line indicates genomic background. (PDF) [file pgen.1007559.s004.pdf]

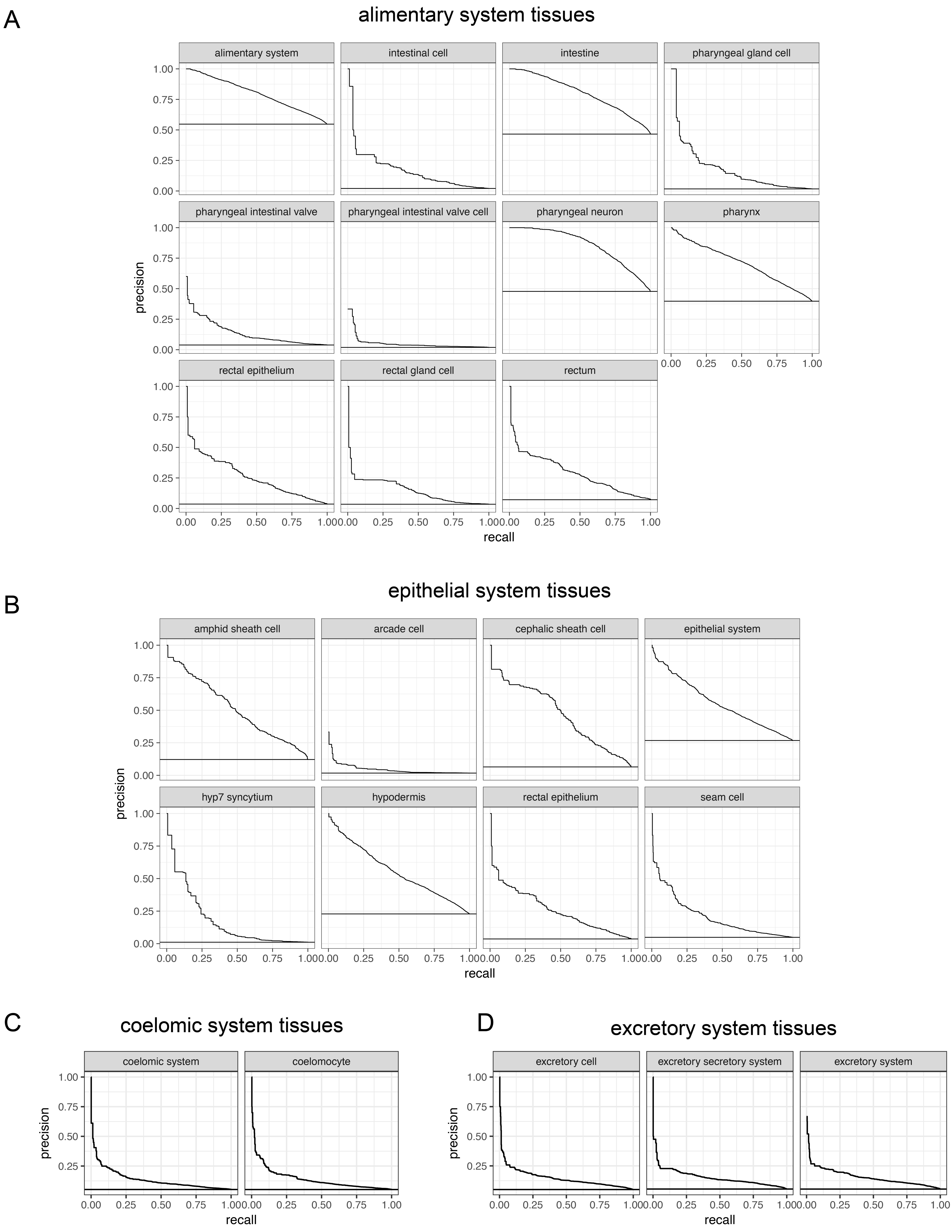

Supplemental Figure 4
